# Are forest disturbances amplifying or canceling out climate change-induced productivity changes in European forests?

**DOI:** 10.1088/1748-9326/aa5ef1

**Published:** 2017-03-16

**Authors:** Christopher P O Reyer, Stephen Bathgate, Kristina Blennow, Jose G Borges, Harald Bugmann, Sylvain Delzon, Sonia P Faias, Jordi Garcia-Gonzalo, Barry Gardiner, Jose Ramon Gonzalez-Olabarria, Carlos Gracia, Juan Guerra Hernández, Seppo Kellomäki, Koen Kramer, Manfred J Lexer, Marcus Lindner, Ernst van der Maaten, Michael Maroschek, Bart Muys, Bruce Nicoll, Marc Palahi, João HN Palma, Joana A Paulo, Heli Peltola, Timo Pukkala, Werner Rammer, Duncan Ray, Santiago Sabaté, Mart-Jan Schelhaas, Rupert Seidl, Christian Temperli, Margarida Tomé, Rasoul Yousefpour, Niklaus E Zimmermann, Marc Hanewinkel

**Affiliations:** 1 Potsdam Institute for Climate Impact Research, Telegrafenberg, P.O. Box 601203, 14412 Potsdam, Germany; 2 Forest Research, Northern Research Station, Roslin, Midlothian, EH25 9SY, United Kingdom; 3 Dept. of Landscape architecture, Planning and Management, Swedish University of Agricultural Sciences (SLU), P.O. Box 66, 230 53 Alnarp, Sweden; 4 Forest Research Centre, School of Agriculture, University of Lisbon, Tapada da Ajuda, 1349-017 Lisboa, Portugal; 5 Forest Ecology, Institute of Terrestrial Ecosystems, Department of Environmental Systems Science, ETH Zurich, Zurich, Switzerland; 6 BIOGECO, INRA—Univ. Bordeaux, Talence, France; 7 Forest Sciences Centre of Catalonia (CTFC-CEMFOR), Ctra. de St. Llorenç de Morunys, km 2, 25280 Solsona, Spain; 8 UMR 1391 ISPA, INRA, Bordeaux Sciences Agro, F-33140 Villenave d’Ornon, France; 9 Department de Biologia Evolutiva, Ecologia i Ciències Ambientals, Universitat de Barcelona. Av. Diagonal 643, 08028, Barcelona, Spain; 10 CREAF. Campus de Bellaterra Edifici C, 08193, Cerdanyola del Vallès, Spain; 11 University of Eastern Finland, School of Forest Sciences, P.O. BOX 101, FI-80101 Joensuu, Finland; 12 Wageningen University and Research Centre, 6700AA, Wageningen, The Netherlands; 13 Institute of Silviculture, Department of Forest and Soil Sciences, University of Natural Resources and Life Sciences, Peter Jordan Straße 82, 1190 Vienna, Austria; 14 European Forest Institute, Yliopistokatu 6, 80100 Joensuu, Finland; 15 Institute of Botany and Landscape Ecology, University of Greifswald, Soldmannstr. 15, 17487 Greifswald, Germany; 16 European Forest Institute, Mediterranean Regional Office (EFIMED), Sant Pau Historic Site, Sant Leopold Pavilion, Carrer St. Antoni M. Claret 167, 08025 Barcelona, Spain; 17 Department of Earth & Environmental Sciences, University of Leuven, Celestijnenlaan 200E box 2411, 3001 Leuven, Belgium; 18 Swiss Federal Research Institute for Forest, Snow and Landscape Research WSL, Landscape Dynamics, 8903 Birmensdorf, Switzerland; 19 Chair of Forestry Economics and Forest Planning, University of Freiburg, Tennenbacherstr. 4, 79106 Freiburg, Germany

**Keywords:** fire, forest models, forest productivity-disturbances-climate change interactions, insects, storms, trade-offs

## Abstract

Recent studies projecting future climate change impacts on forests mainly consider either the effects of climate change on productivity or on disturbances. However, productivity and disturbances are intrinsically linked because 1) disturbances directly affect forest productivity (e.g. via a reduction in leaf area, growing stock or resource-use efficiency), and 2) disturbance susceptibility is often coupled to a certain development phase of the forest with productivity determining the time a forest is in this specific phase of susceptibility. The objective of this paper is to provide an overview of forest productivity changes in different forest regions in Europe under climate change, and partition these changes into effects induced by climate change alone and by climate change and disturbances. We present projections of climate change impacts on forest productivity from state-of-the-art forest models that dynamically simulate forest productivity and the effects of the main European disturbance agents (fire, storm, insects), driven by the same climate scenario in seven forest case studies along a large climatic gradient throughout Europe. Our study shows that, in most cases, including disturbances in the simulations exaggerate ongoing productivity declines or cancel out productivity gains in response to climate change. In fewer cases, disturbances also increase productivity or buffer climate-change induced productivity losses, e.g. because low severity fires can alleviate resource competition and increase fertilization. Even though our results cannot simply be extrapolated to other types of forests and disturbances, we argue that it is necessary to interpret climate change-induced productivity and disturbance changes jointly to capture the full range of climate change impacts on forests and to plan adaptation measures.

## 1 Introduction

In the 20th century, forest productivity in Europe has increased (Spiecker *et al* 1996, Boisvenue and Running 2006). Simultaneously, damage from disturbances, i.e. discrete events destroying forest biomass, has increased as well (Schelhaas *et al* 2003, Seidl *et al* 2014). Both trends are partly associated with a changing climate (Boisvenue and Running 2006, Seidl *et al* 2011), and future projections mostly agree on continued changes in forest productivity (Wamelink *et al* 2009, Reyer *et al* 2014) and disturbances (e.g. Lindner *et al* 2010, Seidl *et al* 2014) due to ongoing climate change.

However, with a few, recent exceptions (e.g. Zubizareta Gerendiain *et al* 2017) most studies projecting future climate change impacts on forests usually only consider either the effects of climate change on productivity (e.g. Kellomäki *et al* 2008, Wamelink *et al* 2009, Reyer *et al* 2014, Reyer 2015) or on disturbances (e.g. Jönsson *et al* 2009, Bentz *et al* 2010, Westerling *et al* 2011, Subramanian *et al* 2015). However, both forest productivity and susceptibility to disturbances change dynamically over forest development as affected by environmental (climate, site) conditions (Urban *et al* 1987, Gower *et al* 1996, Ryan *et al* 1997, Netherer and Nopp-Mayr 2005, Peltola *et al* 2010, Thom *et al* 2013, Hart *et al* 2015).

Furthermore, productivity and disturbance are intrinsically linked: 1) disturbances directly affect forest productivity, e.g. through a reduced ability of the ecosystem to capture resources (e.g. lowered leaf area) or a decreased ability to utilize them (Peters *et al* 2013), and 2) disturbance susceptibility is often coupled to a specific development phase of the forest (Dale *et al* 2000, White and Jentsch 2001), and productivity determines the time a forest remains in this specific phase of susceptibility. For example, the probability of wind damage is strongly associated with tree height and species (Peltola *et al* 1999, Cucchi *et al* 2005, Gardiner *et al* 2010, Albrecht *et al* 2012, Zubizareta Gerendiain *et al* 2017), and forests that are more productive may reach critical heights earlier, increasing their susceptibility to wind damage (Blennow *et al* 2010a, 2010b). In the case of forest fires, it is widely accepted that an increase of productivity implies a higher rate of fuel build-up and subsequently higher fire hazard. However, in managed, even-aged forests, younger, denser forest stands are more susceptible to forest fires (González *et al* 2007, Botequim *et al* 2013, Marques *et al* 2012) and higher productivity may enable them to grow out of this susceptible state faster (Schwilk and Ackerly 2001, Fonda 2001, Keeley *et al* 2011).

Here we compare the ‘*climate-related productivity change*’ (CPC), i.e. the change in forest productivity induced solely by climate change over a specific time period relative to a baseline period, to the ‘*climate- and disturbance-related productivity change’ (CDPC)*, i.e. the change in forest productivity resulting from the joint effects of climate change and disturbances over the same time period relative to a baseline period including disturbances. The objective of this paper is to provide an overview of forest productivity changes in different forests in Europe under climate change, and partition these changes into effects induced by climate change alone and by climate change and disturbances.

**Figure 1:**
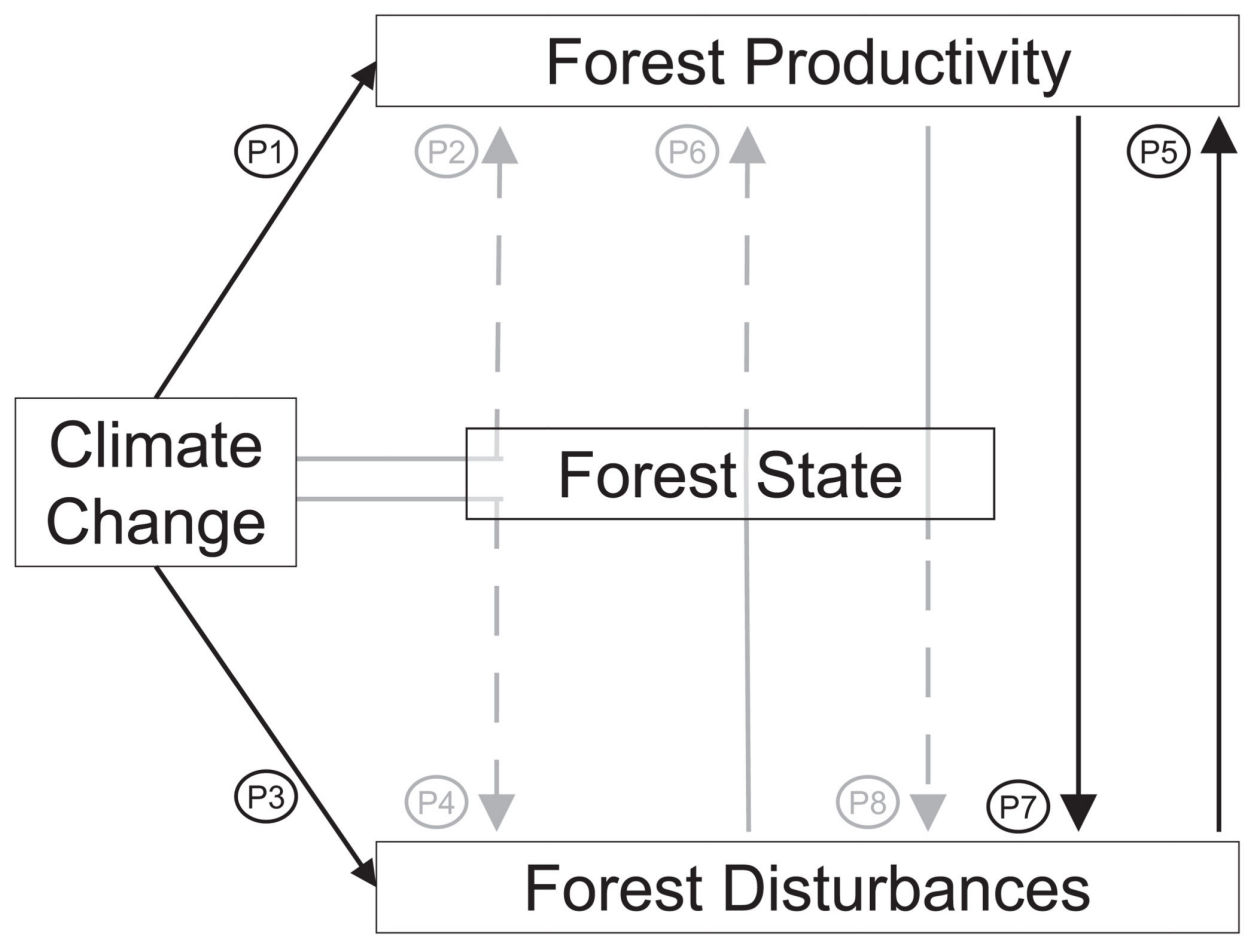
Conceptual framework of interactions between climate change, forest productivity and forest disturbances. Solid, black arrows indicate direct effects; dashed arrows in gray indicate indirect effects mediated through effects on the state of the forests. P1–P8 refer to interaction pathways described in the text.

We present projections of CPC and CDPC from state-of-the-art forest models (table 1) that dynamically simulate forest productivity and the main European disturbance agents (fire, storm, insects), driven by the same climate scenario in seven forest case studies over a large climatic gradient throughout Europe. We classify these models based on a conceptual framework of different pathways of forest productivity-disturbances-climate change interactions (figure 1, table 2) and use them to test how climate change-induced productivity changes are interacting with simultaneously changing disturbances.

## 2 Conceptual framework of forest productivity-disturbances-climate change interactions

Conceptually, the interaction between climate change, forest productivity and disturbances can take eight pathways (P1–P8 in the following) which we characterize as ‘direct’ if the interaction is established through a clear cause-effect relationship while we use ‘indirect’ if the interaction is mediated through changes in the forest state (figure 1). According to this logic, the influence of climate change on productivity and disturbances can take four pathways (P1–P4) just like the interaction between forest productivity and disturbances (P5–P8).

A changing climate *directly* influences key productivity processes such as photosynthesis or respiration (Ryan 1991, Bonan 2008) (P1), but has also *indirect* effects through changes in soil characteristics or changes in species composition (Bolte *et al* 2010) (P2). In turn, disturbances may be *directly* affected by climate change, e.g. through higher wind speeds and changing storm tracks (Shaw *et al* 2016) or higher temperatures increasing bark beetle reproduction rates (Wermelinger and Seifert 1999, Mitton and Ferrenberg 2012) (P3), but could also experience *indirect* effects such as increasing susceptibility to wind damage because of unfrozen soils (Kellomäki *et al* 2010) (P4).

Likewise, disturbances may *directly* influence forest productivity by killing trees (e.g. Michaletz and Johnson 2007) or through more subtle effects of disturbances on productivity (P5). For example, insect defoliation may reduce the amount of absorbed photosynthetic active radiation, the carbon uptake, the stored carbohydrates and nitrogen remobilization, thus reducing overall productivity (Pinkard *et al* 2011) and stem growth (Jacquet *et al* 2012, 2013). Disturbances may also *indirectly* influence forest productivity by changing forest structure and composition (Bolte *et al* 2010, Perot *et al* 2013) (P6). For example, a disturbance-induced increase in tree species diversity can bolster forest productivity (Silva Pedro *et al* 2016). Productivity may also *directly* affect the susceptibility to disturbances (P7). For example, more productive trees may be more vital and hence better able to cope with insect attacks due to an increased availability of carbohydrates for defense (Wermelinger 2004, McDowell *et al* 2011). Changing productivity e.g. due to changing atmospheric CO_2_ concentrations may also influence leaf element stoichiometry and hence influence the palpability and nutritional value of leaves for herbivores (Ayres and Lombardero 2000, Netherer and Schopf 2010). Finally, changing productivity *indirectly* determines a forest’s susceptibility to disturbances by altering key structural features of a forest (P8). For example, simulation studies indicate that increasing productivity under climate change in Sweden leads to increasing height growth and tree heights which in turn increases the probability of wind damage (Blennow *et al* 2010a, 2010b).

## 3 Material and methods

The seven forest case studies studied here are located in North Karelia (Finland), North Wales (United Kingdom), the South-east Veluwe (The Netherlands), Black Forest (Germany), Montafon (Austria), Prades (Spain) and Chamusca (Portugal). They provide a wide range of ecosystem services to society, are shaped by different climatic, edaphic and socio-economic environments and are characterized by varying disturbance regimes (table 1, SOM1 (available at stacks.iop.org/ERL/12/034027/mmedia) cf. Fitzgerald and Lindner 2013, Reyer *et al* 2015). In each case study a specific forest model or differing chains of forest models were applied, utilizing the best available models for each system, and building on a large body of work on testing and evaluating these models for the respective ecosystems. We chose to use the best locally available models for each case study rather than a one-size-fits-all model in order to best capture the local ecosystem dynamics and disturbances, management legacies, species choices and responses to climate change. Consequently, the time periods analyzed and output indicators are not fully homogenized to account for constraints of respective models and local data availability (table 1, see SOM2 for details).

For each forest, four model simulations were carried out: one under baseline climate (B) and one including the effects of climate change on forest productivity (CC) to calculate CPC. Subsequently, these two simulations were repeated also accounting for the effects of disturbances (abbreviated BD and CCD respectively) to calculate CDPC. According to the framework developed in section 3, the simulations required to calculate CPC include the pathways P1 and/or P2 while the simulations for CDPC potentially include all pathways (P1–P8) if included in the model used in each case study (table 2). The climate change simulations all used forcing from the A1B emission scenario from the ENSEMBLES project (van der Linden and Mitchell 2009), and were bias-corrected and downscaled to the respective case study at a 100 m spatial resolution (Zimmermann 2010). All simulations assumed business-as-usual management (two different ones in the Prades region) typical for the region, and expressed changes in productivity using slightly different indicators such as net primary production or mean annual growth, depending on the model applied. More details about the forests, modeling approaches and data sources can be found in table 1 and SOM1-2. In the following, we briefly describe how, in each forest, productivity and disturbances are affected by climate change, following the conceptual framework outlined above (table 2). We then synthesize results from the case studies across the different indicators of forest productivity and disturbances used in each study by comparing CPC and CDPC.

### 3.1 Influence of climate change on productivity and disturbances in the European forest case studies

#### 3.1.1 North Karelia (FI)

In the MONSU simulation system, climate change impacts on productivity were simulated by adjusting species- and site-specific growth functions with data from simulations by a physiological model (Pukkala and Kellomaki 2012). Under a changing climate, the probability of wind damage was expected to increase by 0.17% per year to account for an increase of the unfrozen soil period (Kellomäki *et al* 2010), but no change in wind climate was assumed (Gregow 2013). Productivity changes alter the dominance of different tree species, stocking (stand density), height and height/diameter ratio of trees all of which affect the critical values of wind speed that determine wind damage.

#### 3.1.2 North Wales (UK)

In the ‘MOTIVE8’ model framework (Ray *et al* 2015), temperature, precipitation and moisture deficit affect forest growth. Climate change impacts on forest biomass production were simulated through species- specific scaling of site index. A changing growth rate affects the age at which the trees become vulnerable to windthrow. There was no clear signal of climate change on wind climate in this region, hence the same wind climate as for the past was assumed.

#### 3.1.3 South-east Veluwe (NL)

In the ForGEM model (Schelhaas *et al* 2007), climate change impacts on productivity were mimicked through species-specific scaling of site index according to simulations with a physiological model (Reyer *et al* 2014), see also (Schelhaas *et al* 2015). Since the parameters of the height growth curve are linked to the site class, increasing productivity also means an increase in height growth leading to higher susceptibility to wind damage. There was no clear signal of climate change on wind climate in this case study, hence the historic wind climate was used.

#### 3.1.4 Black forest (GER)

In the LandClim model, temperature and precipitation affect productivity according to response functions and through changes in species dominance (Schumacher *et al* 2004). Changes in temperature affect the reproduction rate of bark beetles. Moreover, bark beetle disturbances depend on drought-stress, age and basal area share of Norway spruce as well as on windthrown spruce biomass (Temperli *et al* 2013). They lead to changes in bark beetle population dynamics. Moreover, LandClim accounts for the beetle-outbreak-triggering effect of windthrow by increased forest susceptibility to bark beetles in the vicinity (<200 m) of windthrow patches and in relation to the windthrown spruce biomass (Wichmann and Ravn 2001). For the simulations considered in this study, the frequency of and area of stochastically simulated windthrow events was assumed to remain constant under climate change, while bark beetles responded dynamically to a changing climate.

#### 3.1.5 Montafon (AT)

In the PICUS v1.5 model, temperature and precipitation affect productivity according to a radiation use efficiency model of stand growth as well as through changes in species dominance (Lexer and Hönninger 2001, Seidl *et al* 2005, Seidl *et al* 2007). Changes in temperature also affect the reproduction rate of bark beetles. Moreover, the bark beetle susceptibility of Norway spruce stands depends on stand age, basal area, host tree share, and drought stress of potential host trees (Seidl *et al* 2007).

#### 3.1.6 Prades (ESP)

In the GOTILWA+ model (Gracia *et al* 1999), temperature and precipitation affect productivity by changing the photosynthetic carbon uptake. Climate change affects the predicted annual fire occurrence probability and fuel moisture. Moreover, drought-stressed trees with reduced amounts of mobile carbohydrates are more likely to die after fire. Changes in productivity modify forest structure and fuel loads and therefore also fire occurrence and severity since the probability of fire is estimated each year, according to the state of the forest (stand basal area, mean and degree of evenness of tree size) and the climatic conditions affecting fuel moisture. Once a fire occurs, it causes mortality plus a temporal (1–3 years) decrease in tree growth (Valor *et al* 2013). The decrease in tree growth can be compensated by ash fertilization or a ‘thinning from below effect’ of fire, depending on fire intensity and structure of the stand. The ‘thinning from below effect’ is in most cases a result of low to medium severity fires (non-stand-replacing fires) that modify stand structure and may reduce tree competition for water resources.

#### 3.1.7 Chamusca (PT)

In the Glob3PG model (Tomé *et al* 2004), temperature and precipitation affect productivity directly through modification of canopy quantum efficiency and, in the case of precipitation, by affecting available soil water that controls biomass allocation to roots. Climate change was assumed to lead to 5% decrease in fire return interval and 5% increase in area burnt.

## 4 Results

### 4.1 Climate change impacts on forest productivity with and without including effects of disturbances

**Figure 2:**
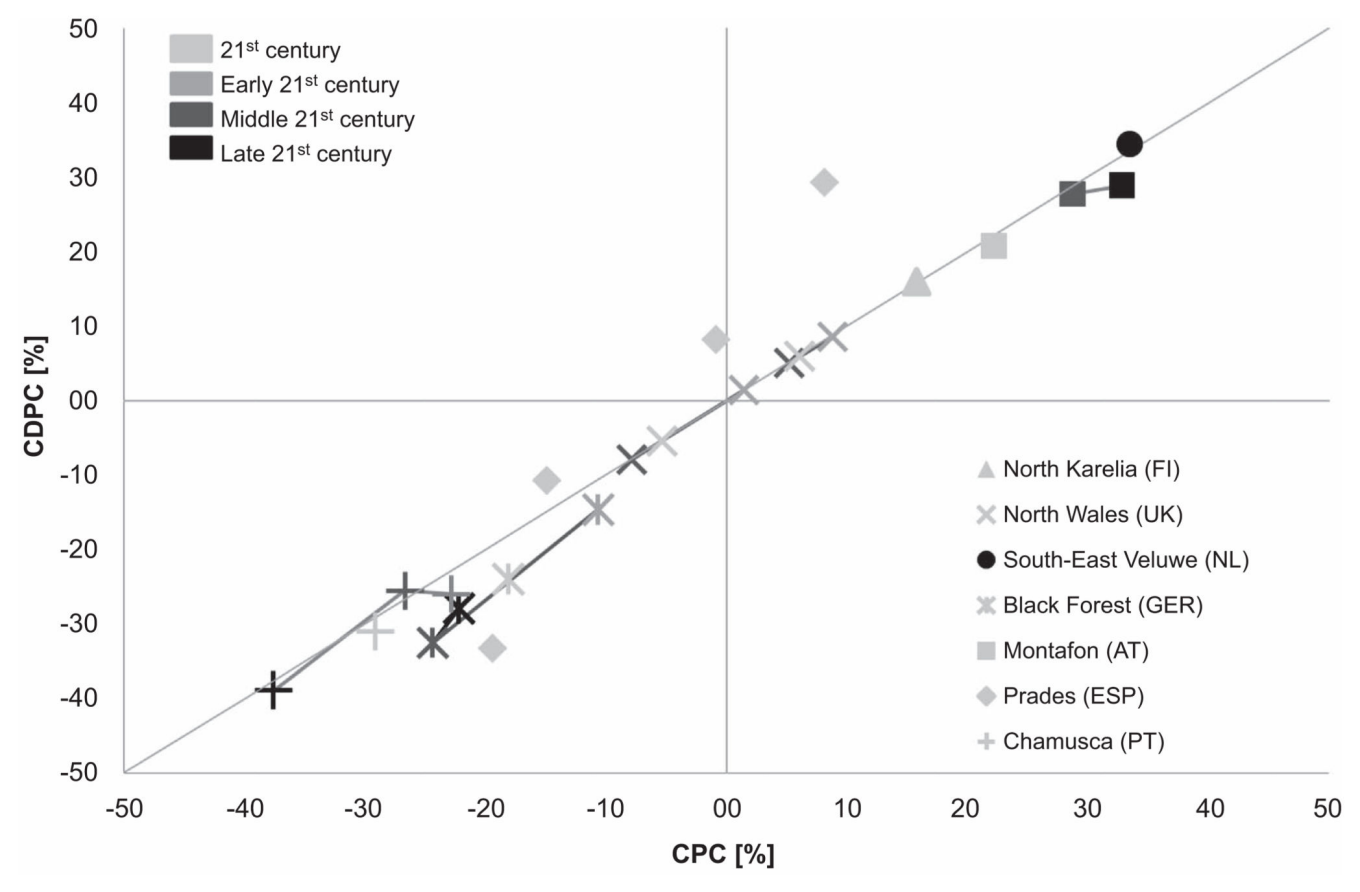
Relative climate change-induced productivity changes with (CDPC) and without (CPC) accounting for disturbances in different forest case studies in Europe. Legend details: 21st century = long-term average over the entire 21st century, Early 21st century = early 21st century average (ca 2000–2040), Middle 21st century = mid-21st century average (ca 2040–2070), Late 21st century = late 21st century average (ca 2070–2100). The exact dates vary slightly according to the different models and are listed in table SOM2. Symbols linked by lines indicate a temporal sequence of results. The horizontal and vertical lines indicate ‘no change’ and the diagonal line is a 1:1 line. Points above the 1:1 line indicate increased productivity as a result of disturbance, while points below it illustrate cases where disturbances decrease productivity.

In North Karelia, South-East Veluwe and Montafon, CPC ranged from +15.8% to +33.6% (figure 2, table SOM2). The productivity increases in North Wales were smaller and turned negative for the drier site. In the Black Forest, CPC was negative and ranged between −10.6% and −24.4%, depending on the time period considered. In the two southern European forest case studies, CPC was mostly negative (−22.8% to −37.6% in Chamusca and −0.8% to −19.4% in Prades) with the exception of forests on deep soils in the Prades region, which showed a small productivity increase (figure 2).

These patterns remained largely consistent when disturbances were included in the simulations (figure 2) with the exception of simulations for the unmanaged Prades forest on deep soils. This forest’s CDPC amounted to +8.2% opposed to a slightly negative CPC (−0.8%) because positive feedbacks from fire caused a release from competition and a fertilization effect.

However, even if the patterns remained the same in most cases, including disturbances had negative effects on productivity, either by reducing positive CPCs or by exacerbating negative CPCs (figure 2). These decreases were rather small and range between −0.05% and −14.0%. In a few cases, including disturbances in the simulations increased positive CDCs but only in the managed Prades forest on deep soils this amounted to a tangible change of +21.1%. In some of the simulations for Prades (unmanaged forest on deep soils and managed forest on shallow soils) and Chamusca (simulation for 2041–2070) regions the negative climate change effects were partly alleviated by including disturbances. These positive effects of disturbances ranged between +1.1% to +9.0%.

For those simulations for which the effects of climate change and disturbances on productivity were studied for more than two time periods, interesting temporal patterns emerged. In the Black Forest, mid-century CDPC was lowest while in Chamusca, the mid-century CDPC was slightly higher than the early- or late 21st century simulations.

**Figure 3:**
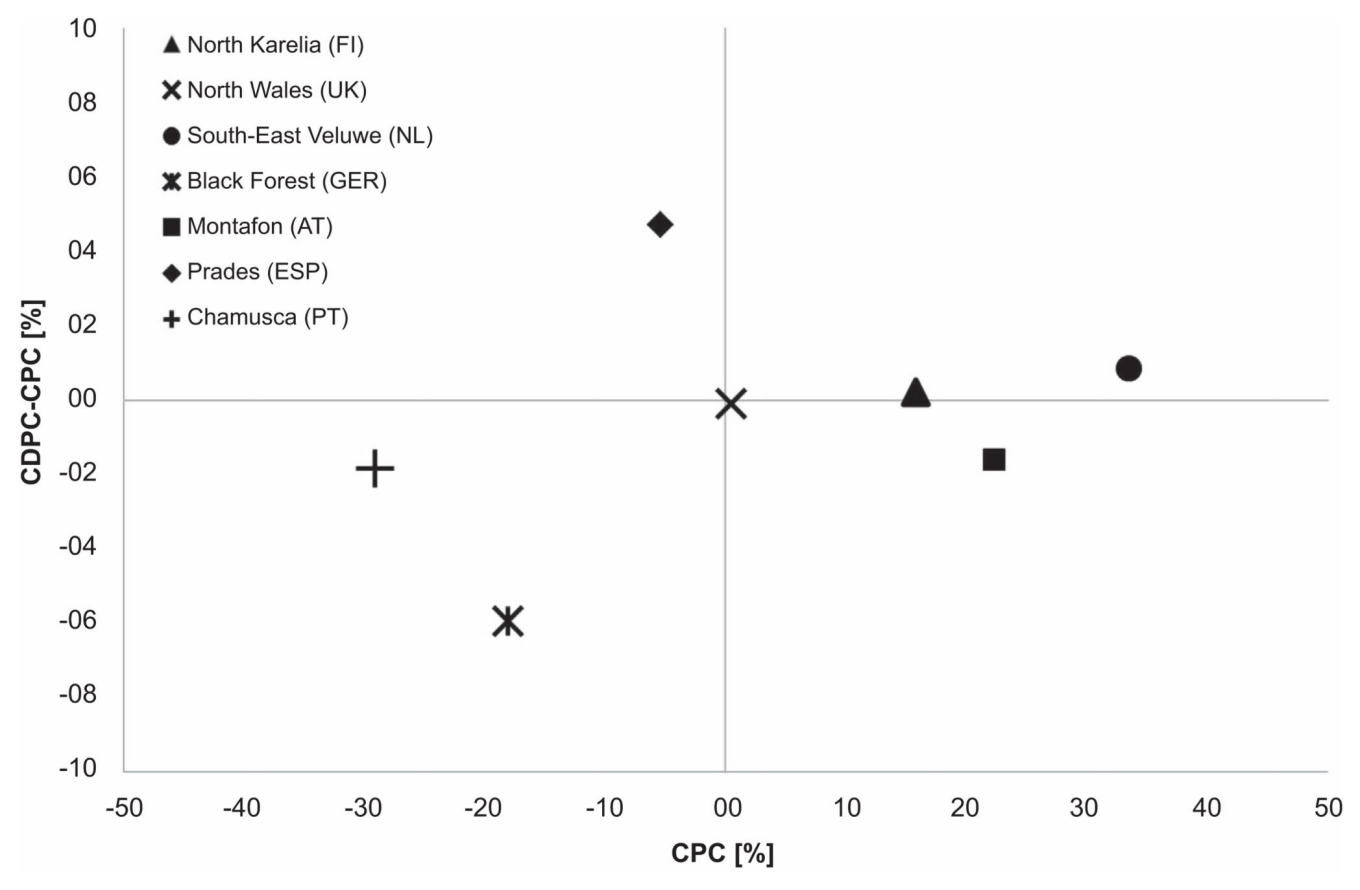
Difference of productivity change induced by climate change and disturbances (CDPC) and climate change only induced productivity changes (CPC) over climate change only induced productivity changes (CPC) for the longest available simulations in each forest case study. Note that the data for Prades and North Wales are the average over the forests stands as shown in table SOM2.

To further test how CPC and CDPC interact, we only considered the difference of CPC and CDPC of those data points that represent the longest possible simulation period for each forest case study (figure 3). This analysis showed that in those forests where CPC was negative (left quadrants in figure 3, Chamusca and Black Forest), disturbances were exacerbating productivity losses. In Prades, disturbances alleviated productivity losses even though the CDPC remained negative. For North Wales and Montafon for which CPCs were positive (right quadrants in figure 3), disturbances were decreasing the positive CPCs but the CDPC remained positive. For the Southern Veluwe and North Karelia, the CDPC was slightly positive because the storm damage in these forests reduced competition among the remaining trees.

## 5 Discussion

This paper shows that climate change-induced productivity changes and disturbances interact in different forests in Europe. In most cases, including disturbances in the simulations clearly exaggerate ongoing productivity declines or cancel out climate change-induced productivity gains. In fewer cases and in some regions only, disturbances also increase productivity or alleviate climate-change induced productivity losses. Only in rather specific situations such as for Prades, they are a real ‘game changer’, turning a climate change-induced productivity loss into a productivity gain. However, in general, the contribution of disturbances to productivity changes compared to those induced by climate change alone is rather small. It is important to note though, that our focus on productivity means that we base the interpretation of our findings on long-term averages (Blennow *et al* 2014) while the higher variability that comes with increased disturbances (as an unplanned event) might still increase management complexity in the short term. Even though this study does not allow us to quantify the individual contribution of the different productivity-disturbances-climate change interaction pathways, we show that indeed such interactions are operating in very different forests across Europe.

### 5.1 Climate change impacts on forest productivity with and without including effects of disturbances

The general trends of increasing CPC in North Karelia, South-East Veluwe and Montafon turning negative if water supply is limited such as in North Wales found in this study are consistent with climate impacts reported in earlier modelling studies for temperate and boreal forests (see Reyer 2015). The rather strong productivity decrease in the Black Forest can be explained by the dominance of Norway Spruce plantations that are very susceptible to climate change (Hanewinkel *et al* 2010, 2013). The decreases in productivity in the two southern European forest case studies (Chamusca and Prades) are also consistent with other modelling studies from Southern Europe (Sabaté *et al* 2002, Schröter *et al* 2005).

Our results reveal interesting temporal patterns of CDPC. The mid-century peak in negative CDPC in the Black Forest region can be explained by two mechanisms: 1) at this time, most of the forest is in a susceptible stage and 2) the damage is so high that later, even though the climate change signal is stronger, less forest area is actually damaged. The combined effects of climate change and bark beetle disturbance lead to a replacement of the beetle’s host species Norway spruce with deciduous and more drought adapted tree species. Similar processes have been found to influence the projected long-term carbon stocks in Swiss forests (Manusch *et al* 2014). Moreover, when considering only the longest possible simulation period for each forest region, the negative, additional effect of disturbances is rather small (maximum −5.9% in the Black Forest, figure 3) which is remarkable given the strong changes in forest composition and structure as well as ecosystem services provision going along with such changes (Temperli *et al* 2012, 2013).

### 5.2 Direct and indirect pathways of productivity-disturbance interactions under climate change

The classification of the models based on the conceptual framework of climate-productivity-disturbance interactions (figure 1) demonstrates that most models are representing both direct and indirect effects of disturbances on productivity (P5–P6, table 2). These models also include indirect effects of changes in productivity on disturbances (P8). However, no model covers all possible pathways and especially the direct effects of changes in productivity on disturbances are not explicitly represented in the set of models used here (P7), possibly because these models do not necessarily operate at the level of process detail required to capture these direct effects, e.g. by excluding leaf element stoichiometry or the role of carbohydrates in plant defense. Moreover, the models mostly cover one or two processes per pathway even though there might be more (e.g. bark beetle reproduction is affected by temperature in LandClim and PICUS but other climatic factors such as drought also play a role (Netherer and Schopf 2010). As our knowledge of these effects evolves the inclusion of such processes into forest models will become more important in the future. It is also important to note that some of the models used in this study also include ‘adaptive management responses’. The management changes according to the disturbance-productivity interactions under climate change by optimizing management to maintain stable resource flows (in Chamusca) or by reducing harvesting age to lower wind risks (in North Wales). More systematic studies of the effect and potential of management interventions to alleviate the effects of changing climate and disturbance regimes on forest productivity are hence needed.

Moreover, there is evidence for many more direct and indirect pathways of productivity-disturbance interactions beyond the ones discussed here (Seidl *et al* 2012). These will require attention in future model applications. Likewise, future studies should also focus on disentangling the importance of the different pathways and their spatial and temporal interactions. Furthermore, it is important to note that disturbances can have a wide variety of other impacts on forests and the services they provide for society beyond changing productivity (Andersson *et al* 2015, Thom and Seidl 2016, Zubizarreta-Gerendiain *et al* 2017).

### 5.3 Limitations and uncertainties

One key limitation of our study is that we are relying only on one emission scenario from one climate model in each of the forest case studies, even though climate impacts differ in between emission scenarios and within emission scenarios when different climate models are considered (Reyer *et al* 2014). Therefore, our simulations do not provide a systematic assessment of the uncertainties induced by climate models and future socio-economic development, but rather provide a first look into how climate change, disturbances and productivity changes are interacting. Moreover, the simulation results presented in this study focus on one main disturbance agent in each forest region to be affected by climate change even though forest productivity may be strongly affected by the occurrence of multiple, compounding and interacting disturbances (Radeloff *et al* 2000, Dale *et al* 2001, Bigler *et al* 2005, Hanewinkel *et al* 2008, Temperli *et al* 2013, Temperli *et al* 2015). Wind-blown or drought-stressed trees for example provide breeding material for insects that then may even attack fully vigorous trees (e.g. Schroeder and Lindelöw 2002, Gaylord *et al* 2013). Newly created forest edges after a storm may expose formerly rather protected trees to subsequent storms. Thus, understanding the spatial and temporal interaction of disturbances and their interaction with changing productivity is another important research challenge (Andersson *et al* 2015, Seidl and Rammer 2016). Moreover, the models used in each forest case study are quite different in the way in which they incorporate the effects of climate change on productivity, and also their representation of disturbances. Therefore, comparing the impacts across different forests can only be done qualitatively, keeping in mind the differences in the models. Moreover, the forest case studies are themselves very different in terms of forest management, species choice etc which are all factors that determine the influence of climate change. Altogether, this means that more variation of the changes in forest productivity under climate change and disturbances than expressed by our results is to be expected. However, our results provide first indications of how climate change and disturbances may play out at larger spatial scales around our forest case studies and similar forest ecoregions.

Finally, this study has focused on the role of disturbances in particular. Future studies should aim at testing the interactions of all pathways of our conceptual framework to gain a full understanding of forest productivity-disturbances-climate change interactions. This could be achieved by developing and applying improved models of disturbance interactions based on experiments and observations of such interactions. Moreover, it would be necessary to study in greater depth whether our findings are consistent over different types of disturbances, stages of stand development, management regimes and soil conditions (which have proven to be very important in e.g. Prades). Such developments could then be integrated into larger-scale simulation models allowing upscaling from the case study level to the continental scale. However, it is important to consider that such larger–scale models will be limited in terms of the number of disturbances and potential interactions that can be included whenever the disturbances are not only resulting from large-scale driving forces (such as extreme heat events depending on planetary waves (Petoukhov *et al* 2016)) but also contingent on local site and forest conditions.

## 6 Conclusion

While the extrapolation of our case study-based results to other types of forests and disturbances requires caution, we argue that our findings have important implications for the assessment of climate change impacts on forest products and services in Europe. On the one hand, higher productivity in a future that is characterized by increasing disturbances may mean that more damage to forests may occur, especially if accompanied by higher standing volume stocks. On the other hand, reduced productivity may mean that less biomass is ‘available to be damaged’ but also that what is damaged is more valuable from a resource availability perspective. Therefore, it is necessary to interpret climate change-induced productivity and disturbance changes jointly to capture the full range of climate change impacts on forests and to plan adaptation. Likewise, these findings are important since currently many model studies, also those relying on models operating at larger spatial scales up to the global level, show that higher productivity will result in higher carbon storage and hence continued carbon uptake from the atmosphere even though the role of disturbances is only cursorily accounted for in many models.

## Supplementary Material

Supplementary material for this article is available online

Supplementary Data

## Figures and Tables

**Table 1:** Key characteristics of the forest case studies. NTFP = Non-timber forest products.

Country	Region	Area	Disturbance	Main ecosystem services	Tree species	Productivity Variable	Models	References introducing the forest region
Finland	North Karelia	950 ha	Wind	Timber, Bioenergy, Recreation, Biodiversity, NTFPs	*Picea abies, Pinus sylvestris, Betula pendula, Betula pubescens*	Mean Annual Timber Yield (m^3^ ha^−1^ yr^−1^)	Monsu^a^	Zubizarreta-Gerendiain *et al* (2016, 2017)
UK	North Wales	11500 ha	Wind	Timber, Recreation, Biodiversity	*Picea sitchensis, Picea abies, Pinus sylvestris, Betula pubescens, Pseudotsuga menziesii, Pinus contorta, Larix kaempferi, Quercus petraea*	Biomass production (t ha^−1^ yr^−1^)	MOTIVE8 simulation using ESC, ForestGALES^b^	Ray *et al* (2015)
Netherlands	South-East Veluwe	1 ha (typical stand)	Wind	Conservation of natural and cultural history, Timber, Recreation	*Pseudotsuga menziesii*	Mean Annual Growth (m^3^ ha^−1^ yr^−1^)	ForGEM^c^, mechanical windthrow module based on HWIND^b^	Hengeveld *et al* (2015), Kramer *et al* (2006)
Germany	Black Forest	1260 ha	Bark Beetle	Timber, Biodiversity, Recreation	*Picea abies, Fagus sylvatica, Abies alba, Pseudotsuga menziesii, Quercus petreae, 25 others.*	Biomass production (t^−1^ ha^−1^ yr^−1^)	LandClim^d^	Temperli *et al* (2012, 2013)
Austria	Montafon	215 ha	Bark Beetle	Timber, Protection	*Picea abies, Abies alba, Fagus sylvatica, Acer pseudoplatanus, Sorbus aucuparia, Alnus incana, Alnus alnobetula*	Net Primary Production (kgC^−1^ ha^−1^ yr^−1^)	PICUS v1.5^e^	Maroschek *et al* (2015)
Spain	Prades	4 typical stands, 1 ha each	Fire	Small-scale forestry, Recreation, NTFPs	*Pinus sylvestris*	Net Primary Production (Mg^−1^ ha yr^−1^)	GOTILWA+ f and adjusted fire model^g^	Sabaté *et al* (2002)
Portugal	Chamusca	483 ha	Fire	Pulp and Paper	*Eucalyptus globulus*	Current Annual Growth (m^3^ ha^−1^ yr^−1^)	Glob3PG^h^ and management optimizer^i^	Palma *et al* (2015)

a Pukkala (2004), Heinonen *et al* (2009), Zubizarreta-Gerendiain *et al* (2017).

b Peltola *et al* (1999), Gardiner *et al* (2000), Nicoll *et al* (2015).

c Schelhaas *et al* (2007).

d Schumacher *et al* (2004, 2006).

e Lexer and Hönninger (2001), Seidl *et al* (2005, 2007).

f Gracia *et al* (1999).

g González *et al* (2006, 2007).

h Tomé *et al* (2004).

i Garcia-Gonzalo *et al* (2014), Rammer *et al* (2014).

**Table 2:** Classification of the models used in this study according to the productivity-disturbances-climate change interaction pathways specified in the conceptual framework shown in figure 1.

Model	Climate change effect on productivity		Climate change effect on disturbances		Disturbance effect on productivity		Productivity effects on disturbance	

	*Direct (P1)*	*Indirect (P2)*	*Direct (P3)*	*Indirect (P4)*	*Direct (P5)*	*Indirect (P6)*	*Direct (P7)*	*Indirect (P8)*
Monsu	Species- and site-specific scaling of growth functions/site index according to simulations with physiological model	Change in species composition	Na	Probability of wind damage increases by 0.17% per year due to gradual increase of unfrozen soil period	Wind damage reduces forest productivity when windthrown trees are not harvested	Non-optimal harvesting time may reduce forest productivity via effects on forest structure	Na	Changes in dominance of different tree species, stocking (stand density), height and height/diameter ratio of trees.
MOTIVE8	Temperature, precipitation and moisture deficit affect growth	Na	Na	Na	Wind damage before planned harvest date reduces forest productivity	Harvesting before stands reach Maximum Mean Annual Increment to reduce wind risk reduces forest productivity as the full productive potential of the site is never reached	Na	Changes in height growth alter susceptibility to wind damage
ForGEM + mechanical windthrow module based on HWIND	Species- and site-specific scaling of growth functions/site index according to simulations with physiological model	Na	Na	Na	Removal of trees	Effect on forest structure	Na	Changes in height growth alter susceptibility to wind damage
LandClim	Temperature and precipitation affect growth	Change in species composition	Changes in temperature affect the reproduction rate of bark beetles	Bark beetle disturbance susceptibility depends on drought-stress, age and basal area share of Norway spruce as well as the windthrown spruce biomass	Bark beetle disturbance causes tree mortality decreasing forest productivity	Change in species composition	Na	Basal area share of Norway spruce influences bark beetle disturbance susceptibility
PICUS v1.5	Temperature, precipitation, radiation and vapor pressure deficit affect growth	Temperature and precipitation affect tree species composition	Changes in temperature affect the reproductive rate of bark beetles	Bark beetle susceptibility depends on drought stress of host trees as well as host tree availability, basal area, and age	Disturbances reduce leaf area and thus the radiation absorbed, which in turn affects productivity	Change in species composition	Na	Stand structure (age, Norway spruce share) influences bark beetle disturbance susceptibility
GOTILWA + and adjusted fire model	Temperature and precipitation affect growth	Na	Climate change affects the predicted annual fire occurrence probability	Drought-stressed trees are more susceptible to die after fire	Mortality and a temporal (1 to 3 years) decrease in tree growth	Ash fertilization; a ‘thinning from bellow effect’ of fire reducing competition for water	Na	Probability of fire and post-fire mortality are estimated according to the structure of the forest
Glob3PG and management optimization method	Temperature and precipitation affect growth	Na	Climate change leads to 5% decrease in fire return interval and 5% increase in area burnt	Na	Increased fire frequency and increased affected area destroy biomass	Periodical reductions in area productivity due to fire, changes optimum management in each management unit attempting to respect flow constraints	Na	Na
