# Supplementary material for: Are forest disturbances amplifying or canceling out climate change-induced productivity changes in European forests?
Source: Environ Res Lett. Author manuscript; Available in PMC 2017 Aug 27. (PMC5572643; doi:10.1088/1748-9326/aa5ef1)
Supplement: Supplementary Data [file NIHMS73653-supplement-Supplementary_Data.pdf]

Supporting Online Material (SOM) for:

# Are forest disturbances canceling out or amplifying climate change-induced productivity changes in European forests?

Christopher P.O. Reyer[1], Stephen Bathgate[2], Kristina Blennow[3], Jose G. Borges[4], Harald Bugmann[5], Sylvain Delzon[6], Sonia P Faias[4], Jordi Garcia-Gonzalo[4,7], Barry Gardiner[2,8], Jose Ramon Gonzalez-Olabarria[7], Carlos Gracia[9,10], Juan Guerra Hernández[4], Seppo Kellomäki[11], Koen Kramer[12], Manfred J. Lexer[13], Marcus Lindner[14], Ernst van der Maaten[15], Michael Maroschek[13], Bart Muys[16], Bruce Nicoll[2], Marc Palahi[14], João HN Palma[4], Joana A Paulo[4], Heli Peltola[11], Timo Pukkala[11], Werner Rammer[13], Duncan Ray[2], Santiago Sabaté[9,10], Mart-Jan Schelhaas[12], Rupert Seidl[13], Christian Temperli[17,5], Margarida Tomé[4], Rasoul Yousefpour[18], Niklaus E. Zimmermann[17,5], Marc Hanewinkel[17,18]

[1]Potsdam Institute for Climate Impact Research, Telegrafenberg, P.O. Box 601203, 14412 Potsdam, Germany

[2]Forest Research, Northern Research Station, Roslin, Midlothian, EH25 9SY

[3]Dept. of Landscape architecture, Planning and Management, Swedish University of Agricultural Sciences (SLU), P.O. Box 66, 252 30 Alnarp, Sweden

[4]Forest Research Centre, School of Agriculture, University of Lisbon, Tapada da Ajuda, 1349-017 Lisboa, Portugal

[5]Forest Ecology, Institute of Terrestrial Ecosystems, Department of Environmental Systems Science, ETH Zurich, Zurich, Switzerland

[6]BIOGECO, INRA - Univ. Bordeaux, Talence, France

[7]Forest Sciences Centre of Catalonia (CTFC-CEMFOR), Ctra. de St. Llorenç de Morunys, km 2, 25280 Solsona, Spain

[8]UMR 1391 ISPA, INRA, Bordeaux Sciences Agro, F-33140 Villenave d'Ornon, France

[9]Department de Biologia Evolutiva, Ecologia i Ciències Ambientals, Universitat de Barcelona. Av. Diagonal 643, 08028, Barcelona, Spain.

[10]CREAF. Campus de Bellaterra Edifici C, 08193, Cerdanyola del Vallès, Spain.

[11]University of Eastern Finland, School of Forest Sciences, P.O. BOX 101, FI-80101 Joensuu, Finland

[12]Wageningen University and Research Centre, 6700AA, Wageningen, The Netherlands

[13]Institute of Silviculture, Department of Forest and Soil Sciences, University of Natural Resources and Life Sciences, Peter Jordan Straße 82, 1190 Vienna, Austria

[14]European Forest Institute, Joensuu Finland

[15]Institute of Botany and Landscape Ecology, University of Greifswald, Soldmannstr. 15, 17487 Greifswald, Germany

[16]European Forest Institute, Mediterranean Regional Office (EFIMED), Sant Pau Historic Site, Sant Leopold Pavilion, Carrer St. Antoni M. Claret 167, 08025 Barcelona, Spain
[17]Swiss Federal Research Institute for Forest, Snow and Landscape Research WSL, Landscape Dynamics, 8903 Birmensdorf, Switzerland.
[18]Chair of Forestry Economics and Forest Planning, University of Freiburg, Tennenbacherstr. 4, 79106 Freiburg, Germany

# SOM 1: Descriptions of the forest regions

This section introduces the main challenges for forest management, expected climate change impacts on forests and disturbances in the different case study regions. Moreover a brief description of the climate, assumed climate change, soils and forest stands used in this study are provided.

## *North Karelia (Finland)*

### General description

The forests in the Finnish case study area are traditionally used for timber production. This is still the main use, but the production of energy biomass is increasing in the form of harvesting logging residues, including stumps (Finnish Statistical Year Book of Forestry 2014). However, also recreation (landscape values), maintenance of biodiversity and now-wood values (berries, mushrooms, moose hunting etc) are considered in forest management in this area, depending on the interests of forest owners. In the case study area, the changing climate may in general enhance forest productivity (Kellomäki et al. 2008) but also tree mortality due to high wind speeds and/or heavy snow loading with shorter duration of soil frost from late autumn to early spring (Päätalo et al. 1999; Peltola et al. 1999; Kellomäki et al. 2010; Peltola et al. 2010; Gregow et al. 2011). In summer storms, especially Norway spruce (*Picea abies*) but also Scots pine (*Pinus sylvestris*) and birch (*B. pendula* and *B. pubescens*) (in leaf) are vulnerable to wind damage, whereas in winter time Norway spruce with shallow rooting is the most vulnerable (Peltola et al. 2010). The mortality may also increase due to drought episodes and especially in Norway spruce with shallow rooting on sites with relatively low water-holding capacity (Kellomäki et al. 2008). Pests and pathogens are also expected to become more aggressive under the warming climate (e.g. *Ips typographus* and *Heterobasidion*).

### Climate, soil and stand description

The current climate (average of 1960 -2000) used in this study was that of the Joensuu airport (N 62° 40', E 29° 38', asl 94 m). Under changing climate, $CO_2$ increased from 363 ppm to 635 ppm. Annual mean temperature increased from 2 to 6 °C, and summer mean temperature (June, July, August) increased from 14 to 17 °C. Annual precipitation increased from 500 mm to 550 mm, summer precipitation remaining nearly the same as currently.

The study area is 950 ha, representing the managed forests dominated by even-aged stands of Scots pine (*Pinus sylvestris*), Norway spruce (*Picea abies*) and birch (*B. pendula* and *B. pubescens*). A part of land (44%) is of low fertility occupied by Scots pine, whereas 32% of land is of medium fertility occupied all the species alone or in mixtures. The rest (24%) of land is of high fertility occupied mainly by Norway spruce and birch alone or in mixtures. In the study area, 914 ha of total land area is of productive one (the annual mean growth ≥ 1 $m^3$ $ha^{-1}$ $yr^{-1}$), distributed in 650 separate stands (see Zubizarreta Gerendiain et al. 2016). Scots pine represents 53%, Norway spruce 31% and birch 16% of total stocking of stem wood. Age class distribution is even, representing 25, 39.4, 19.5 and 16.1% of classes 0-19 yr, 20-59 yr, 60–99 yr and >100 yr. At the beginning of the simulation, the predicted mean annual volume growth per hectare was 4.3 m3/ha on productive forest land area.

## *North Wales (United Kingdom)*

## General description

Two publically owned forests managed by Natural Resources Wales (formerly Forestry Commission Wales), namely Gwydr and Clocaenog, were studied (Ray et al.2014). The forests are similar in size, Clocaenog is approximately 5662 hectares while Gwydr is 5839 hectares. In addition both forests were established over a short time span in response to afforestation initiatives in the 20th century. This has led to a narrow distribution of age classes and restructuring of the forests is a priority.

## Climate, soil and stand description

The forests differ considerably in their bioclimatic site types, purpose and species diversity. Clocaenog is an upland forest with nutrient poor soils and greater wind exposure, optimized for productive forestry. The average annual temperature in Clocaenog is 7.2°C and annual precipitation 1194mm. In contrast Gwydr contains more sheltered site types and richer soils, allowing a wider range of species to be planted. Despite being situated to the west of Clocaenog, the majority of Gwydr forest is at lower elevations and the annual temperature is 9.1°C and annual precipitation 987mm. Gwydr is also utilized to a greater extent for multipurpose forestry objectives such as recreation and biodiversity.
The main species planted in Clocaenog is Sitka spruce which occupies around 73% of the planted area, with Norway spruce (14%), Larch species (6%) and other species forming the remainder. Gwydr, through the better site conditions, has a greater proportion of other species, Sitka spruce is still dominant at 50%, but broadleaves cover 9% of the area, with other conifers such as Douglas fir, Scots pine and Lodgepole pine occupy the remainder. Most of those stands are stocked initially at densities between 2000-4000 stems per hectare, with thinning being more common in Gwydr. Although most stands were established around even aged monocyclic principles there is increasing interest in continuous cover forestry in both study areas.
Climate projections (Murphy et al. 2009) indicate a likelihood of warmer, drier conditions. Although warmer conditions might be expected to improve growth rates, we expect precipitation to increase in the winter and to decline in the growing season, leading to higher moisture deficits from 2050 onwards which would reduce the growth potential of spruce (Ray et al. 2015).

## *South-East Veluwe (The Netherlands)*

Moderate changes in climate are not expected to lead to major problems or changes in the area (Hengeveld et al. 2015). Drier and hotter summers are expected to lead to a decline in oak and beech forests and increased dominance of coniferous species like Scots pine and Douglas fir. The combination of a shift to coniferous species and drier and hotter summers is expected to lead to increased fire risk. The Netherlands has been hit by a few destructive storms in the past, but damage has been small over the last 2 decades, despite an increase in growing stock and forest area (Schelhaas et al. 2014). However, it is still located in the usual range of storm tracks and future storm occurrence is inevitable. Increased productivity might lead to more risk due to taller trees and increased growing stocks. It is still uncertain how the future storm climate will develop.

## Climate, soil and stand description

Weather variables such as daily radiation, temperature and precipitation are generated by a weather generator, assuming the stand to be located in the middle of the South-East Veluwe area. Average annual temperature is 9.4°C and annual precipitation 850mm. For wind climate we used the same climate as replicate number 1 of the climate in Schelhaas (2008), indicative for the wind climate at measurement station Leeuwarden (53°13' N, 5°46' E) (KNMI, 2007). Soil quality is translated in the model by defining a site class for the site under study. For current climate conditions, we used site class 12 for Douglas fir according to Janssen et al. (1996). Simulations are done with an initial stand of 10-year old Douglas fir trees, 4000 stems per ha, planted in a regular pattern, with an average diameter of 7cm and a height of 5.7m.

## *Black Forest (Germany)*

The case study is located in the northern Black Forest in south-west Germany (48°40' N, 8°13' E) and is represented by a 1260 ha (ca. 2 by 7 km) elevation transect that encompasses 500–1000 m a.s.l. As in large parts of Central Europe past forest management heavily promoted Norway spruce by planting and thinning, which led to Norway spruce dominated forests also beyond the hot-dry end of its natural distribution range. Forest stability and the provision of timber and biodiversity related ecosystem goods and services by these Norway spruce forests are considered to be threatened by climate change-induced drought, bark beetle disturbances and windthrow. Forest managers and researchers now recommend adaptive forest management to convert these forests to more drought-adapted and disturbance-resistant, mixed forests (Spiecker et al. 2004) e.g. in admixture with European beech (Yousefpour and Hanewinkel, 2014).

## Climate, soil and stand description

Current climate is oceanic with mean annual temperature (1950–2000, 845 m a.s.l.) being 7.0°C and annual precipitation 1095mm. Climate change projections for 2081-2100 provided by the general circulation model and regional climate model run HadCM3Q0/HadRM3Q0 by the Hadley Center for Climate Prediction and Research based on the IPPC AR4 A1B emission scenario indicate a temperature increase  4.6°C. Projected changes in annual precipitation are with –4% relatively minor while larger changes of –17% are expected for the growing season (Apr-Sep; see also Temperli et al. 2013). Soils in the case study landscape are intermediately drained with soil water holding capacities between 6–15 cm. Past management led to a mosaic of even-aged, almost pure Norway spruce stands of varying age (map of forest stands and soil water capacity provided by Forstliche Versuchsanstalt Baden-Württemberg, cf. Temperli et al. 2012).

## ***Montafon Valley (Austria)***

The Austrian case study is located at the border to Switzerland, situated in an alpine landscape called the Montafon. The forests in the region are strongly dominated by Norway spruce (*Picea abies*) and silver fir (*Abies alba*) with some admixed European beech (*Fagus sylvatica*), sycamore maple (*Acer pseudoplatanus*), ash (*Fraxinus excelsior*), Scots pine (*Pinus sylvestris*), European larch *(Larix decidua*) as well as some other less abundant coniferous and deciduous tree species. Most of the forests are uneven-aged and located on steep slopes that require skyline based logging techniques. Ecosystem services demanded at local and regional level are primarily timber production (saw logs and fuelwood) and protection against gravitational natural hazards, such as snow avalanches, rock fall, landslide, debris flow, erosion, and flooding.
The anticipated future climate shows a distinct increase in temperature (Lindner et al. 2010). No major changes are expected for precipitation (Zimmermann 2010). Tree growth and reproduction potential is expected to benefit tree species in the high altitudes of the Montafon valley because of more favorable growing conditions and longer growing seasons (Lindner et al. 2010). However, for Norway spruce the risk of bark beetle infestations will also increase substantially due to more favourable development conditions for poikilothermal insects. Especially in the second half of the 21st century a strong increase in bark beetle induced mortality can be expected. Bark beetles are the by far most important biotic disturbance agent in the Montafon, abiotic disturbances such as windthrow and snowbreak are only of minor importance.

### Climate, soil and stand description

The current climate is temperate (oceanic) with annual mean temperatures of 4°C and an average annual precipitation of 1220 mm at 1500 m a.s.l. (the case study spans from 1160 to 1800 m a.s.l.). The anticipated climate change projects a distinct increase in temperature of 3.7°C (scenario A1B) at the end of the $21^{st}$ century and no major changes are expected for precipitation (based on CCLM/ECHAM5 data, for more information please see Zimmermann, 2010). The dataset has a spatial resolution of 100x100 m over a period of 100 years from 2001 to 2100 featuring daily values.
The geology in the northern part of the case study area is characterized by calcareous bedrock (limestone and dolomite) whereas in the larger southern part crystalline bedrocks (e.g. gneiss and amphibolite) dominate. On calcareous bedrock rendzinas, luvisols and cambisols are dominating. Ranker, podsols, semi-podsols and shallow cambisols are the most important soils on crystalline parent rock material. Top-soil profiles have been made during the filed survey in the year 2010 to derive soil characteristics.
The stand characteristics for the 215 ha case study have been collected during the field season in 2010 by using a sample plot approach. In combination with LiDAR-data (Hollaus et al., 2006 and 2007) realistic mountain forest structures have been derived for the initialization of simulation entities in PICUS v 1.5. The stands can be characterized as uneven-aged. Norway spruce accounts for 67.3% of the basal area, silver fir for 14.3%, European beech for 8.3% and the remaining 10% are consisting of other broadleaves like sycamore maple, rowan (*Sorbus aucuparia*) and alders (*Alnus incana* and *A. alnobetula*).

## *Prades (Spain)*

In the Catalonian case study, we used the GOTILWA+ simulation tool to assess the effect of climate change and forest management on wood production in a forest stand of Scots pine (Pinus sylvestris) located in the Muntanyes de Prades. It is a coastal mountain range near Tarragona with a sub-mediterranean climate; it has an isolated semi-natural population of Scots pine at the southern margin of its distribution range. After the abandonment of the traditional forest management (mainly oak coppice for charcoal making) in the second half of the last century, the area received very little forest management. Today´s forest has a high density and a high growing stock, is fire-prone, and shows indications of forest decline.
The simulations consider the current climate and one climate change scenario, two different soil types, and two management options. The study also considers the possibility of fire affecting the stand and killing a portion of the growing stock. By integrating fire risk into GOTILWA+ it is possible to analyze the combined effect on the forest of the two main hazards for the Mediterranean forest, drought and forest fires. All climate change scenarios point to a decreasing precipitation in Southern Europe and an increase in the frequency and severity of dry spells. Moreover, climate change is expected to prompt variations in the fire regime across the region, causing an increment in the number of large forest fires.

### Climate, soil and stand description

The climate scenario A1B projected by a general circulation model and a regional model (ECHAM4/AEMET) was used to run 100 years of simulation. This scenario assumed an increase of 2.1 ppm/year in $CO_2$ concentration, an increase of mean annual temperature by 6.5°C, a precipitation decrease of 30%, and a potential evapotranspiration increase of 7% over the next 100 years.
The simulations were run starting from a young even-aged stand of *Pinus sylvestris*, with an initial density of 2308 trees/ha and a mean dbh of 6.62 cm, mimicking the typical structure of young stands in *Prades*. The two representative soils used in the simulations were a shallow soil (52 cm depth) with a water holding capacity of 107.7mm (A03) and a deep soil (148 cm depth) with a water holding capacity of 306.4 mm (A82).

## *Chamusca (Portugal)*

The Portuguese case study is located in the region of Chamusca, approx 100 km NE of Lisbon. Forestland extends over 70% of the territory. Cork oak (*Quercus suber* L.) and Eucalypt (*Eucalyptus globulus* Labill.) pure stands are the main forest cover types extending over 58% of the area. While cork oak stands are managed for the extraction of cork, the eucalyptus plantations are managed to supply eucalypt fiber to pulp mills.
Contrary to central and northern Europe where the future climate is expected to lead to an increase of productivity (Lindner et al. 2010), in Portugal the climate is expected to become hotter and drier (Soares et al, 2012) thus leading, in one hand, to a decrease of productivity and, in the other hand, to an increase of fire risk, the most important disturbance in this region. Oliveira et al (2011) analyzed regional fire occurrence patterns and concluded that wildfires have a returning interval of 23 years and that about 0.8% of the area burns every year. We took advantage of these findings, to examine the impact of climate change and fire disturbances in a private industrial property with eucalypt stands extending over 483 ha. Although open to discussion, we considered a future wildfire scenario with a decrease of the fire return interval of 5% and an increase of 5% in annual area burnt.

## Climate, soil and stand description

A brief analysis of the climate scenarios based on WorldClim (Hijmans et al, 2005) were found to be inadequate for local or regionalized case studies in Portugal, also confirming findings by Bedia et al. (2013), probably due to the low number of observed data points to develop WorldClim dataset, increasing the error in extrapolated data, thus becoming inadequate for use at local scale. Therefore, the A1B scenario, sensitivity 3Q0 (normal sensitivity), provided by the regional climate model from the Hadley Centre (Collins et al. 2006), was used instead as it is considered the most appropriate for the Portuguese context (Soares et al. 2012).
The Tejo River, the longest river in the Iberian Peninsula, goes through this region. Chamusca has two distinct areas: Campo, left side of the river with 3 km width and an altitude between 15-25 m; and Charneca that encompasses podzol soils with several water courses, and an altitude between 100-190 m. The industrial property considered in this study lies within the Charneca. The 483 ha eucalyptus forest was classified into 69 and 10 pure and mixed (with cork oak) management units, respectively Because of fire occurrence in years 2003 and 2005, the stands are characterized by a heterogeneous age distribution with a high number of young stands (SOM Figure 1).

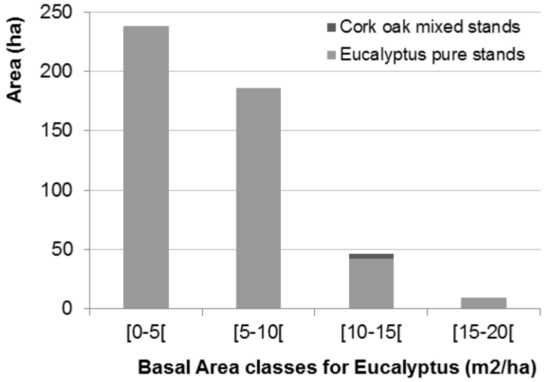


SOM Figure 1: Basal area classes distribution of the eucalyptus stands in the property.

# SOM 2: Model descriptions

## *North Karelia (Finland)*

### General Model description

The MONSU optimizing system considers several objective variables such as timber production, carbon balance of forestry (carbon in forest biomass and soil and in wood products), biodiversity (dead wood) and risks of damages due to high wind speeds and extreme snow loading (Heinonen et al. 2009). The MONSU optimising system was made sensitive to climate change by introducing climate change-induced growth trends. These trend were based on simulations with a process-based ecosystem model as described by Pukkala and Kellomäki (2012). The trends in diameter growth due to changing climate are species- and site-specific and hence could be expected to affect optimal management solutions (Zubizarreta Gerendiain et al. 2015).

### Simulation approach

We employed the MONSU simulation-optimization system combined with the growth trend related to changing climate in order to study how changing productivity interacts with wind-induced mortality and how this affects the predicted productivity. Simulations were done at the management unit level over 60 years for the period 2012-2072. The baseline management followed the current recommendations for privately-owned forests.

We simulated the development and management of each stand over 60 years, using a five-year time step. The simulation design included the simulations both under current climate and changing climate (A1B scenario), with and without considering the effects of wind damages on forest growth. Critical wind speeds (m $s^{-1}$) for uprooting were calculated for each stand based on approach used by Heinonen et al. (2009). Critical values depend on tree species, mean height/diameter ratio of trees and relative wind shelter given by neighbor stands. Each stand was assumed to be surrounded by four neighboring stands (heights randomly estimated based on uniform height distribution between 0 and 30 m), with the gap size (diameter if the stand) being fixed as five three heights. Critical value of wind speed was computed only for the edge where the adjacent stand had the smallest tree height.

Probability of wind damage (critical wind speed) was calculated every five years for each stand. In doing this, the occurrence of maximum wind speeds (of at least 10-min duration during a ten-year period) was assumed to be normally distributed in our study area with the measured mean of 13.8 +/-3 m $s^{-1}$ (Heinonen et al. 2009). Under a changing climate, the probability of damage was expected to increase by 0.17% per year since the start of simulation due to gradual increase of unfrozen soil period until 2100 (Kellomäki et al. 2010), but no change in wind climate was assumed (see Gregow 2013). Stochastic wind damage was generated in the stand with the obtained probability. Small damages were expected to be more frequent than severe ones (SOM Figure 2). A percentage equal to the simulated damage rate was removed from the volume of growing stock. It was assumed that wind-thrown trees are not harvested. The total 60-year yields of the four different scenarios were calculated as: Yield = Ending volume - Initial volume + Harvested volume.

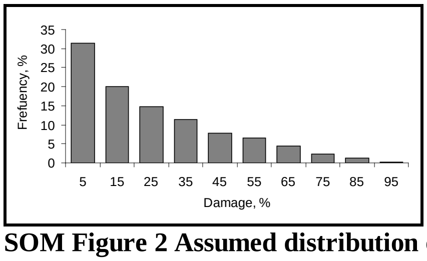


**SOM Figure 2 Assumed distribution of the severity of wind damage.**

## *North Wales (United Kingdom)*

### General Model Description

The ‘MOTIVE8’ model framework (Ray et al. 2014) provides an analysis of ecosystem service indicators coupled with risk metrics related to forest productivity and the risk to damaging storms. The framework integrates climate sensitive site yield potential models and growth models, which operate at the stand scale. Outputs from growth models provide input data for models that predict outputs of provisioning, regulating and cultural services. The stand level yield projections are also utilized with a national scale wind climate map to assess the wind risk of a stand at a given moment in time. Assessments of wind risk were based on the assumption that each forest management unit had a wind-firm edge. Operational digital stock maps are utilized as input data, as there are many potential combinations of species, management and site type to consider.

### Simulation Approach

Two contrasting forest areas in North Wales, Clocaenog and Gwydr forests, were simulated using the ‘MOTIVE8’ approach. These forests differ in altitude and species composition (see Ray et al. 2014), with Gwydr being lower altitude and more diverse then Clocaenog. Yield and changes to yield with climate data from baseline to 2080 were modelled using Ecological Site Classification (ESC) (Pyatt et al. 2001). Climate data required by ESC was input into the simulation using all variants of the 11-member-RCM data set corresponding with an A1B emissions scenario in UKCP09 (Murphy et al. 2009). Disturbance was modelled as wind damage using the ForestGALES model (version 2.5) (Hale et al. 2015, Nicoll et al. 2015).
The biomass increment of a forest management unit (e.g. subcompartment) was calculated yearly and presented as a yearly per hectare average for each reporting period. To assess potential disturbance losses, when the percentage probability of damage from ForestGALES was below 1% we assumed there was no effect, otherwise the disturbed biomass was calculated as a percentage of the management unit biomass. Stand scale results were then aggregated and reported at forest level. Analyses were conducted for the baseline period, and for all 11 variants of the RCM, although data based on the 11 variants were averaged for the purpose of this paper. The assumed forest management strategy was to replace species, like for like, following harvesting. Strong cyclical influences upon production levels remain at both study areas owing to large scale forest establishment being conducted over a short time period last century. This trend continued in the simulation due to application of 50 year rotation lengths for production stands. Data were aggregated to forest-scale based on production and disturbance in individual sub-compartments from the “Sub-compartment database” (Forestry Commission / NRW) that is used to manage the public forest estate.

## *South-East Veluwe (The Netherlands)*

### General Model description

ForGEM (Forest Genetics, Ecology and Management) is a forest model that simulates the growth and development of individual trees, on a scale up to several hectares. For each tree age, height, stem diameter at breast height (dbh), crown length and radius, and mass of foliage, branches, heartwood, sapwood, coarse roots and fine roots are tracked over time. Tree growth is driven by light interception. In the gap type approach, light is assumed to come straight from above. The light interception in each 20 m × 20 m grid cell is divided over the trees in the cell, according to their foliage mass and its vertical distribution (see (Bugmann 2001)). Intercepted radiation is converted into photosynthates which are allocated in such a way that specific ratios between different tree compartments are maintained (see Kramer et al. 2008; Kramer and van der Werf 2010). Photosyntates allocated to the stem are converted into height and dbh increment. Maximum height increment follows a Richards' growth curve ((Richards 1959; Jansen 1996). Actual height increment can be lower due to insufficient resources. The relationship between tree volume, height and dbh is described by species-specific allometric functions, derived from yield table data (Jansen 1996). Resource related mortality appears when all reserves of a tree are depleted and there is no foliage left. Age related mortality is assumed to follow a two-parameter Weibull distribution.

Schelhaas et al. (2007) added a mechanical windthrow module to the ForGEM model, based on the HWIND approach (Peltola et al. 1993, Gardiner et al. 2000). It takes into account the windload to individual trees, as influenced by surrounding trees via sheltering and support, and collateral damage by falling neighbouring trees.

### Simulation approach

The simulation setup was the same as in Schelhaas (2008), i.e. a core area of 1 ha, surrounded by a 50m buffer on all sides except on the eastern side. Windthrow is only allowed in the core area since the model is not able to deal with acclimation processes, which mainly occurs in exposed edge trees (Cucchi et al. 2004). This version of ForGEM is using the Radiation Use Efficiency (RUE) approach and productivity is thus not directly depending on a changed climate. ForGEM is calibrated to different Douglas fir site classes (Jansen et al. 1996) by adapting the RUE. Here we mimic a changed climate by an increase in site class. As current climate we took site class 12. According to the results by Reyer et al. (2014) in a modelling study with the 4C model, climate change would lead on average to a 27% increase in productivity for Picea abies in Germany (A1B scenario with $CO_2$ sensitivity) in the period 2070-2100. The RUE increases from site class 12 to 16 by 24.3%, and thus we take site class 16 as an indication for growth conditions under climate change conditions. We assume the same stand to be growing throughout its full life in either current or future climate conditions.

Since it is unclear how the wind climate will change under climate change conditions, we applied the same wind climate data for the current climate and future climate runs. All 4 combinations of current climate/future climate and with and without wind were performed, assuming a management along the lines of Douglas fir site class 12. This entails a thinning cycle of 5 years, where trees with the smallest diameter are removed until the desired density is reached (as calculated within 20*20m grid cells).

## *Black Forest (Germany)*

### General Model description

Simulations of forest development and bark beetle disturbance have been conducted with the forest landscape model LandClim (Schumacher et al. 2004, 2006), where forest landscape dynamics are modeled stochastically as a function of climate and soil properties on a grid of 25 × 25 m cells. Within each cell a simplified forest gap model (Bugmann and Solomon 2000) is used to simulate regeneration, growth and mortality of tree cohorts of the same species and size. Disturbance sub-models representing fire, wind, bark beetles and forest management are implemented at the landscape scale (Schumacher and Bugmann 2006, Temperli et al. 2012). Simulated forests and forest processes have been proven to be consistent with empirical data in various applications (Schumacher et al. 2006, Colombaroli et al. 2010, Henne et al. 2011, Elkin et al. 2012, Temperli et al. 2012).
The simulation of bark beetle (*Ips typographus*) dynamics integrates assessments of forest susceptibility to bark beetle infestation (Netherer and Nopp-Mayr 2005, Seidl et al. 2007) and bark beetle phenology (Wermelinger and Seifert 1999, Baier et al. 2007). This approach is employed at the landscape scale to determine the spatial distribution of bark beetle infestation and at the scale of individual cells to determine bark beetle-induced tree mortality. Both beetle infestation and beetle-induced tree mortality are dynamically linked to forest dynamics within the LandClim modeling framework and thus interact with climate, the forest state and other disturbances including windthrow.

### Simulation approach

We ran simulations from 2000 through 2100. Besides using a baseline current climate scenario (1950–2000) we represented climate change projections with a rather strong climate change scenario based on the A1B emission pathway and the GCM/RCM combination HadCM3Q0/HadRM3Q0. In order to provide a baseline comparison, we also simulated forest dynamics without bark beetle disturbance under current climate and under climate change. We assumed a continuation of even-aged Norway spruce management (clearcut at a dominant dbh of >45 cm, replanting of spruce) and no changes in the wind storm regime (for simulations under varying windthrow regimes we refer to Temperli et al. 2013). We initialized the scenarios with simulation data generated by simulating the past management under current climate until a pseudo-equilibrium state was reached and replicated each simulation 15 times to account for the stochasticity inherent to the simulation of disturbances.

## *Montafon Valley (Austria)*

### General Model description

The simulations have been conducted with the hybrid forest gap model PICUS. This model builds on a 3-D structure of 10 x 10 m patches, extended vertically by crown cells of 5 m height. PICUS simulates individual trees and is capable of handling spatially explicit simulation entities (e.g. stand polygons) up to several hectares. Tree population dynamics emerge from growth, mortality and reproduction. A detailed management module allows the simulation of spatially explicit management actions ranging from simple clear cuts to single tree removals. Disturbances from the European Spruce Bark Beetle (*Ips typographus* L. Col. Scol.) are explicitly considered in the model (Lexer and Hönninger, 2001; Seidl et al. 2005; 2007). PICUS v1.5 was used for the simulations.

### Simulation approach

In total 53 simulation entities summing up to 215 ha embedded in a framework of non-stockable gullies and avalanche tracks have been defined. The detailed case study features one south- and one north-facing slope, both ranging from 1160 to 1800 m a.s.l.
A matrix of two climate scenarios (baseline (BL) and A1B) and two disturbance scenarios were simulated. The disturbance related scenarios, one with disturbances by I. typographus (BB) and the second without disturbances (noBB), complete the simulation design matrix. The simulations were conducted for a period of 90 years from 2010 to 2100.
Current management (BAU) has been considered in the simulations. BAU practices rely on long-distance cable yarding with skyline systems. The skyline tracks are set up diagonal along the slopes over distances of up to 1000 m. Along the skyline tracks irregularly shaped slit and patch cuts are implemented in order to initiate and favor natural regeneration. The current management intensity (i.e. implemented skyline tracks per year) equals a virtual rotation length of 250 years.
All occurring species have been considered in assessing the productivity under the four scenarios.

## *Prades (Spain)*

### General Model description

GOTILWA+ (**G**rowth **O**f **T**rees **I**s **L**imited by **Wa**ter) is a process-based forest growth model that simulates physiological processes related to forest growth and explores how these processes are influenced by climate, tree stand structure, management techniques, soil properties and climate change (Gracia et al., 1999).

Fire risk was integrated in GOTILWA+ by estimating for each simulation step the probability of fire occurrence during the simulation step duration and the potential damage once a fire affect a stand. For predicting the probability that a fire occurred during each step of the simulation and the potential post-fire fire damage, expressed in proportion of dead trees, similar models to the ones in Gonzalez et al. (2006) and Gonzalez et al. (2007) were used. However, the new models were adjusted in a way that explicitly considered climatic conditions and water availability, better fitting with GOTILWA+ outputs. Due to the stochastic nature of the fire model, it would be desirable to get the averages of multiple simulations. However, as GOTILWA+ is a process based model and quite complex this is limited by computational power.

### Simulation approach

All simulations started with the same initial stand characteristics and were run for a period of 100 years according to current climate and the A1B climate scenario. Eight simulations were run per time period combining two soil types with management vs. no management, and with no fire risk vs. fire risk. Management implied a management regime with four cutting events at years 10, 40, 71 and 99 of the simulation, the first two operations corresponding to thinning, the third one to a preparatory regeneration cutting where the basal area is reduced to 20 $m^2$/ha, and the last one being a final cut where all remaining trees were removed.

For the "fire included" simulations, which are based on the same stand and management conditions as the "fire free" simulations, GOTILWA+ simulates fires stochastically, based on the predicted annual fire occurrence probability, and trees get killed according to the fire damage model. For each of the "fire included" simulations, various medium intensity fires were generated, differing in number, timing and effect, depending on the soil depth and management regime. For example, for the stand growing under the climate change scenario on shallow soil 3 fires occurred (years 3, 13 and 33 of the simulation) when no management was applied, and 2 fires (years 42 and 95) when management was applied. In the stands growing in deep soils 4 fires occurred if no management was applied (years 43, 45, 50 and 93) and 3 fires when management was applied (years 30, 52 and 59). Each of the simulated fires killed a proportion of the growing stock depending on the stand structure at the moment of the fire occurrence. The dead trees were removed from the simulation and the remaining surviving trees followed their development. Under the current climate scenario, fires were also similarly generated randomly.

## *Chamusca (Portugal)*

## General Model description

The analysis took advantage of two modeling tools. Firstly, the forest model Glob3PG, a hybridization of a process-based model (3PG) with allometric for stand basal area, stand volume and dominant height that link the 3PG biomass outputs with other outputs from existing empirical models (Tomé et al 2004), was used to project the growth of the current inventory in each stand. Secondly, a mathematical model was developed in order to obtain a management plan that maximizes timber production while enforcing a non declining timber yield under climate change scenarios. Simulations with and without fire were also considered. The reader is referred to Garcia-Gonzalo et al. (2013), Rammer et al. (2014) and Palma et al (2015) for a detailed description of this approach.
Briefly, the Glob3PG projects different prescriptions (varying plantation density, harvest age, and coppicing rotations) for each stand. The tabular projections are processed to build a mixed integer programming (MIP) mathematical model that ensures that one, and only one, prescription may be chosen for each stand. A MIP algorithm (e.g. CPLEX©) is used to solve the mathematical problem, i.e. to select a prescription for each stand. The objective function of the MIP model was designed to have a goal programming (GP) format where the goals correspond to the volume needed to be harvested in each planning period. The levels of attainment of each goal were defined by taking the average of the harvest flows in the solution of a MIP unconstrained run. Thus the GP objective function minimized the deviations from this average timber flow in each period.

## Simulation approach

Four scenarios were considered: A) Baseline where current climate is used, B) Baseline with current fire disturbance, C) Future climate and D) Future climate with future fire disturbance (see SOM table 1). The baseline climate is the average of 30 years (1970-2000) and every year of 2010-2100 has the same monthly data of the 1971-2000 timeframe.
The simulations were performed over a 90 year planning horizon (2010-2100) using 4-year periods. Optimization models were used to target the maximum sustainable yield while meeting even-flow of harvests. A preliminary assessment using an unconstrained formulation (i.e. with no even-flow constraints) indicated that the average timber yield over the temporal horizon might reach at the most 30000m3 and 20000m3 in current and future climate scenarios, respectively. The disturbance scenario (wildfires) was designed to reflect the findings by Oliveira et al (2011). It was thus assumed that wildfires would have a returning interval of 23 years and that about 0.8% of the 483 ha would burn every year if no climate change occurs. Future scenarios of fire behavior are difficult to predict. However, for comparison purposes, we considered that future climate would lead to a 5% decrease in the fire return period and to a 5% increase % in the area burnt in each fire event (see SOM table 1).

SOM Table 1: Fire disturbance events modeled in the case study during 2010-2100

| Climate Scenario | Fire Event | 1 | 2 | 3 | 4 |
|---|---|---|---|---|---|
| Current | Year | 2033 | 2056 | 2079 | - |
| | Area Affected (ha) | 90 | 90 | 90 | - |
| Future | Year | 2032 | 2053 | 2072 | 2091 |
| | Area Affected (ha) | 94 | 99 | 104 | 109 |

## SOM 3: Simulation results

SOM Table 2: Key simulation settings and results. B = Baseline; CC = Climate Change, CPC = climate-related productivity change; BD = Baseline+Disturbance; CCD = Climate Change+Disturbances; CDPC = climate- and disturbance-related productivity change. Note that CPC and CDPC are in %, while B, CC, BD and CCD are in the units specified in the column "variable". For further explanations see the main text.

| Region | Baseline | Future Time Period | Variable | B | CC | CPC | BD | CCD | CDPC | GCM/RCM | $CO_2$ change | T change | P change |
|---|---|---|---|---|---|---|---|---|---|---|---|---|---|
| North Karelia | 1960-2001 | 2012-2072 | Mean Annual Timber Yield (m3/ha/yr) | 2.9 | 3.3 | 15.8% | 2.5 | 2.9 | 16.0% | (indirectly via FinnFor runs) | 363 to 635 | 4°C | 50mm |
| North Wales -Gwydr | 1961-1990 | 2011-2040 | Timber production (tonnes/ha/year) | 5.56 | 5.64 | 1.44% | 5.49 | 5.57 | 1.46% | HadRM3 11 member RCM | na | 1.4°C | 0 ( +7 winter, -7 summer) |
| North Wales -Gwydr | 1961-1990 | 2041-2070 | Timber production (tonnes/ha/year) | 5.48 | 5.05 | -7.85% | 5.45 | 5.02 | -7.89% | HadRM3 11 member RCM | na | 2.5°C | -3( +14 winter, -17 summer) |
| North Wales -Gwydr | 1961-1990 | 2011-2080 | Timber production (tonnes/ha/year) | 5.66 | 5.36 | -5.3% | 5.61 | 5.31 | -5.35% | HadRM3 11 member RCM | na | 3.5°C | -1( +19 winter, -20 summer) |
| North Wales - Clocaenog | 1961-1990 | 2011-2040 | Timber production (tonnes/ha/year) | 5.91 | 6.43 | 8.8% | 5.81 | 6.31 | 8.61% | HadRM3 11 member RCM | na | 1.4°C | 0( +7 winter, -7 summer) |
| North Wales - Clocaenog | 1961-1990 | 2041-2070 | Timber production (tonnes/ha/year) | 5.18 | 5.45 | 5.21% | 5.12 | 5.38 | 5.08% | HadRM3 11 member RCM | na | 2.5°C | -3( +14 winter, -17 summer) |
| North Wales - Clocaenog | 1961-1990 | 2011-2080 | Timber production (tonnes/ha/year) | 5.76 | 6.11 | 6.08% | 5.68 | 6.02 | 5.99% | HadRM3 11 member RCM | na | 3.5°C | -1( +19 winter, -20 summer) |
| North Wales (average Gwydr & Clocaenog) | 1961-1990 | 2011-2080 | Timber production (tonnes/ha/year) | 5.7 | 5.7 | 0.44% | 5.6 | 5.7 | 0.35% | HadRM3 11 member RCM | na | 3.5°C | -1( +19 winter, -20 summer) |
| South Veluwe | 1961-2005 | 2071-2100 | Mean Annual Growth (m3/ha/yr) | 10.9 | 14.5 | 33.6% | 10.8 | 14.5 | 34.4% | (indirectly via 4C runs) | indirectly via 4C runs | indirectly via 4C runs | indirectly via 4C runs |
| Black Forest | 1950-2000 | 2001-2100 | Biomass production (t/ha/yr) | 6.6 | 5.4 | -18.1% | 6.0 | 4.6 | -24.0% | HadCM3Q0/HadRM3Q0 | na | 4.2°C | -4% |
| Black Forest | 1950-2000 | 2001-2040 | Biomass production (t/ha/yr) | 6.8 | 6.1 | -10.6% | 6.1 | 5.2 | -14.6% | HadCM3Q0/HadRM3Q0 | na | 1.4°C | -1% |
| Black Forest | 1950-2000 | 2041-2070 | Biomass production (t/ha/yr) | 6.8 | 5.1 | -24.4% | 6.3 | 4.3 | -32.5% | HadCM3Q0/HadRM3Q0 | na | 3.2°C | -8% |
| Black Forest | 1950-2000 | 2071-2100 | Biomass production (t/ha/yr) | 6.1 | 4.7 | -22.2% | 5.7 | 4.1 | -27.9% | HadCM3Q0/HadRM3Q0 | na | 4.2°C | -4% |
| Montafon | 1960-2000 | 2010-2100 | NPP (kgC/ha/yr) | 951.8 | 1164.1 | 22.3% | 953.1 | 1150.4 | 20.7% | ECHAM5/CLM | na | 1.9°C | -1% |
| Montafon | 1960-2000 | 2041-2070 | NPP (kgC/ha/yr) | 960.8 | 1236.9 | 28.7% | 962.5 | 1229.3 | 27.7% | ECHAM5/CLM | na | 1.8°C | 1% |
| Montafon | 1960-2000 | 2071-2100 | NPP (kgC/ha/yr) | 948.1 | 1259.5 | 32.8% | 951.3 | 1226.2 | 28.9% | ECHAM5/CLM | na | 3.7°C | -7.6% |
| Prades (unmanaged, shallow soil) | 1981-1998 | 2000-2100 | NPP (Mg/ha/year) | 4.1 | 3.3 | -19.4% | 5.3 | 3.6 | -33.3% | ECHAM4/AEMET | 2.1 ppm/yr | 6.5°C | -30% |
| Prades (unmanaged, deep soil) | 1981-1998 | 2000-2100 | NPP (Mg/ha/year) | 5.0 | 4.9 | -0.8% | 5.9 | 6.4 | 8.2% | ECHAM4/AEMET | 2.1 ppm/yr | 6.5°C | -30% |
| Prades (managed, shallow soil) | 1981-1998 | 2000-2100 | NPP (Mg/ha/year) | 5.5 | 4.7 | -14.8% | 5.4 | 4.9 | 4.2% | ECHAM4/AEMET | 2.1 ppm/yr | 6.5°C | -30% |
| Prades (managed, deep soil) | 1981-1998 | 2000-2100 | NPP (Mg/ha/year) | 6.4 | 6.9 | 8.2% | 5.9 | 7.6 | 21.1% | ECHAM4/AEMET | 2.1 ppm/yr | 6.5°C | -30% |
| Prades (average) | 1981-1998 | 2000-2100 | NPP (Mg/ha/year) | 5.2 | 4.9 | -5.4% | 5.6 | 5.6 | -0.7% | ECHAM4/AEMET | 2.1 ppm/yr | 6.5°C | -30% |
| Chamusca | 1971-2000 | 2011-2100 | Current Annual Growth (m3/ha/yr) | 15.8 | 11.2 | -29.1% | 15.5 | 10.7 | -31.0% | HadCM3Q0/HadRM3Q0 | na | 2.4°C | -13.1% |
| Chamusca | 1971-2000 | 2011-2040 | Current Annual Growth (m3/ha/yr) | 16.2 | 12.5 | -22.8% | 15.8 | 11.7 | -25.9% | HadCM3Q0/HadRM3Q0 | na | 1.3°C | -7.8% |
| Chamusca | 1971-2000 | 2041-2070 | Current Annual Growth (m3/ha/yr) | 15.0 | 11.0 | -26.7% | 14.5 | 10.8 | -25.5% | HadCM3Q0/HadRM3Q0 | na | 2.4°C | -10.7% |
| Chamusca | 1971-2000 | 2071-2100 | Current Annual Growth (m3/ha/yr) | 16.5 | 10.3 | -37.6% | 16.2 | 9.9 | -38.9% | HadCM3Q0/HadRM3Q0 | na | 3.6°C | -21.2% |

## References

Baier, P., J. Pennerstorfer, and A. Schopf. 2007. PHENIPS—A comprehensive phenology model of Ips typographus (L.) (Col., Scolytinae) as a tool for hazard rating of bark beetle infestation. Forest Ecology and Management 249:171–186. doi: 10.1016/j.foreco.2007.05.020.

Bedia, J., Herrera, S., Gutiérrez, J.M., 2013. Dangers of using global bioclimatic datasets for ecological niche modeling. Limitations for future climate projections. Glob. Planet. Change 107, 1–12. doi:10.1016/j.gloplacha.2013.04.005

Bugmann, H. 2001. A review of forest gap models Climatic Change. 51:1573-1480.

Bugmann, H. K. M., and A. M. Solomon. 2000. Explaining forest composition and biomass across multiple biogeographical regions. Ecological Applications 10:95–114.

Collins, M., Booth, B., Harris, G., Murphy, J., Sexton, D., Webb, M., 2006. Towards quantifying uncertainty in transient climate change. Climate Dynamics 27, 127-147.(datasets available @ www.ensembles-eu.org)

Colombaroli, D., P. D. Henne, P. Kaltenrieder, E. Gobet, and W. Tinner. 2010. Species responses to fire, climate and human impact at tree line in the Alps as evidenced by palaeo-environmental records and a dynamic simulation model. Journal of Ecology 98:1346–1357. doi: 10.1111/j.1365-2745.2010.01723.x.

Cucchi, V., Meredieu, C., Stokes, A., Berthier, S., Bert, D., Najar, M., Denis, A., Lastennet, R., 2004. Root anchorage of inner and edge trees in stands of Maritime pine (Pinus pinaster Ait.) growing in different podzolic soil conditions. Trees – Structure and Function, 18 (4), 460-466.

Elkin, C., B. Reineking, C. Bigler, and H. Bugmann. 2012. Do small-grain processes matter for landscape scale questions? Sensitivity of a forest landscape model to the formulation of tree growth rate. Landscape Ecology 27:697–711. doi: 10.1007/s10980-012-9718-3.

Finnish Statistical Year Book of Forestry. 2014. National Resources Institute Finland. Helsinki. Vammalan Kirjapaino Oy. ISBN 9789514024504.

J. Garcia-Gonzalo, J. G. Borges, J. H. N. Palma, A. Zubizarreta-Gerendiain, 2013,A decision support system for management planning of Eucalyptus plantations facing climate change, Annals of Forest Science, DOI: 10.1007/s13595-013-0337-1

Gardiner, B., H. Peltola, Kellomäki, S., 2000. Comparison of two models for predicting the critical wind speeds required to damage coniferous trees. Ecological Modelling 129 (1), 1-23.

González, J.R., Palahí, M., Trasobares, A., Pukkala, T., 2006. A fire probability model for forest stands in Catalonia (North-East Spain). Annals of Forest Science 63, 169-176.

González, J.R., Trasobares, A., Palahí, M., Pukkala, T., 2007. Predicting stand damage and tree survival in burned forests in Catalonia (North-East Spain). Annals of Forest Science. 64, 733-742.

Gracia, C A, Tello E, Sabaté S and Bellot J 1999 Gotilwa: An integrated model of water dynamics and forest growth. In: F Rodà, J Retana, C Gracia and J Bellot. (Eds.). Ecology of Mediterranean Evergreen Oak Forest. Ecological Studies Vol. 137. Springer-Verlag, Heidelberg, pp. 163–179.

Gregow, H., Peltola, H., Laapas, M., Saku, S., Venäläinen, A. 2011. Combined occurrence of wind, snow loading and soil frost with implications for risks to forestry in Finland under the current and changing climatic conditions. Silva Fennica 45(1): 35-54.

Gregow, H. 2013. Impacts of strong winds, heavy snow loads and soil frost conditions on the risks to forests in Northern Europe. Finnish meteorological Institute Contributions 94: 178 p.

Hale, S.E., Gardiner, B., Peace, A., Nicoll, B., Taylor, P. and Pizzirani, S., (2015). Comparison and validation of three versions of a forest wind risk model. Environmental Modelling & Software, 68, pp.27-41.

Heinonen, T., Pukkala, T., Ikonen, V.-P., Peltola, H., Venäläinen, A., Dupont, S. 2009. Integrating the risk of wind damage into forest planning. Forest Ecology and Management 258(7):1567-1577.

Hengeveld GM, Didion M, Clerkx APPM, Elkin C, Nabuurs GJ, Schelhaas MJ 2015. The landscape level effect of individual-owner adaptation to climate change in Dutch forests. Regional Environmental Change15:1515-1529 DOI: 10.1007/s10113-014-0718-5

Henne, P. D., C. M. Elkin, B. Reineking, H. Bugmann, and W. Tinner. 2011. Did soil development limit spruce (Picea abies) expansion in the Central Alps during the Holocene? Testing a palaeobotanical hypothesis with a dynamic landscape model. Journal of Biogeography 38:933–949. doi: 10.1111/j.1365-2699.2010.02460.x.

Hijmans, R.J., S.E. Cameron, J.L. Parra, P.G. Jones and A. Jarvis, 2005. Very high resolution interpolated climate surfaces for global land areas. International Journal of Climatology 25: 1965-1978.

Hollaus, M., Wagner, W., Eberhöfer, C., Karel, W. 2006. Accuracy of large-scale heights derived from LiDAR data under operational constraints in a complex alpine environment. ISPRS Journal of Photogrammetry & Remote Sensing 60, 323-338.

Hollaus, M., Wagner, W., Maier, B., Schadauer, K. 2007. Airborne Laser Scanning of Forest Stem Volume in a Mountainous Environment. Sensors 7, 1559-1577.

Jansen, J.J., Sevenster, J., Faber, P.J. (Eds.), 1996. Opbrengsttabellen voor belangrijke boomsoorten in Nederland. IBN Rapport 221, Hinkeloord Report No 17.
KNMI, 2007. Dutch Royal Meteorological Institute www.knmi.nl Page assessed at 23 July 2007

Kellomäki, S., Peltola, H., Nuutinen, T., Korhonen, K. T., Strandman, H. 2008. Sensitivity of managed boreal forests in Finland to climate change, with implications for adaptive management. Philosophical Transactions of the Royal Society B: Biological Sciences 363(1501):2341-2351.

Kellomäki, S., Maajärvi, M., Strandman, H., Kilpeläinen, A., Peltola, H. 2010. Model computations on the climate change effects on snow cover, soil moisture and soil frost in the boreal conditions over Finland. Silva Fennica 44(2):213-233.

KNMI 2007 Dutch Royal Meteorological Institute. http://www.knmi.nl (assessed on 27 September, 2016).

Kramer, K., J. Buiteveld, M. Forstreuter, T. Geburek, S. Leonardi, P. Menozzi, F. Povillon, M. Schelhaas, E.T. du Cros, G.G. Vendramin and D.C. van der Werf. 2008. Bridging the gap between ecophysiological and genetic knowledge to assess the adaptive potential of European beech. Ecological Modelling. 216:333-353.

Kramer K.; van der Werf, D. 2010. Equilibrium and non-equilibrium concepts in forest genetic modelling: population-and individually-based approaches. Forest Systems. 3:100-112.

Lexer, M. J. and Hönninger, K. 2001. A modified 3D-patch model for spatially explicit simulation of vegetation composition in heterogeneous landscapes. Forest Ecology and Management, 144, 43-65.

Lindner, M., Maroschek, M., Netherer, S., Kremer, A., Barbati, A., Garcia-Gonzalo, J., Seidl, R., Delzon, S., Corona, P., Kolström, M., Lexer, M.J., Marchetti, M. 2010. Climate change impacts, adaptive capacity, and vulnerability of European forest ecosystems. Forest Ecology and Management, 259(4), 698-709.

Murphy J, Sexton D, Jenkins G, Booth B, Brown C, Clark R, Collins M, Harris G, Kendon E, Betts R, Brown S, Boorman P, Howard T, Humphrey K, McCarthy M, McDonald R, Stephens A, Wallace C, Warren R, Wilby R, Wood R. (2009). UK Climate Projections Science Report: Climate change projections. Met Office Hadley Centre: Exeter.

Netherer, S., and U. Nopp-Mayr. 2005. Predisposition assessment systems (PAS) as supportive tools in forest management--rating of site and stand-related hazards of bark beetle infestation in the High Tatra Mountains as an example for system application and verification. Forest Ecology and Management 207:99–107. doi: 16/j.foreco.2004.10.020.

Oliveira Sofia L. J., Pereira José M. C., Carreiras João M. B. (2011) Fire frequency analysis in Portugal (1975–2005), using Landsat-based burnt area maps. International Journal of Wildland Fire 21, 48-60.  DOI http://dx.doi.org/10.1071/WF10131

Päätalo, M-L., Peltola, H., Kellomäki, S. 1999. Modelling the risk of snow damage to forests under short-term snow loading. Forest Ecology and Management 116:51-70.

Palma, J. H. N., J. A. Paulo, S. P. Faias, J. Garcia-Gonzalo, J. G. Borges, and M. Tomé 2015 Adaptive management and debarking schedule optimization of Quercus suber L. stands under climate change. Case study in Chamusca, Portugal. Regional Environmental Change 15: 1569-1580 http://dx.doi.org/10.1007/s10113-015-0818-x

Peltola, H., Kellomäki, S., 1993. A mechanistic model for calculating windthrow and stem breakage of Scots pines at stand edge. Silva Fennica 27, 99-111.

Peltola, H., Kellomäki, S., Väisänen, H., Ikonen, V-P. 1999. A mechanistic model for assessing the risk of wind and snow damage to single trees and stands of Scots pine, Norway spruce and birch. Canadian Journal of Forest Research 29: 647-661.

Peltola, H., Ikonen, V-P., Gregow, H., Strandman, H., Kilpeläinen, A., Venäläinen, A., Kellomäki, S. 2010. Impacts of climate change on timber production and regional risks of wind-induced damage to forests in Finland. Forest Ecology and Management 260(5):833-845.

Pukkala, T., and Kellomäki, S. 2012. Anticipatory vs adaptive optimization of stand management when tree growth and timber prices are stochastic. Forestry 85 (4): 463-472.

Pyatt, G., Ray, D., and Fletcher, J. (2001). An Ecological Site Classification for Forestry in Great Britain. Bulletin 124, Forestry Commission, Forest Research. www.forestry.gov.uk/pdf/ESCBulletin.pdf/$FILE/ESCBulletin.pdf.

Rammer W, C. Schauflinger, H. Vacik, J. H.N. Palma, J. Garcia-Gonzalo, J. G. Borges and M. J. Lexer 2014 A web-based ToolBox approach to support adaptive forest management under climate change Scandinavian Journal of Forest Research 29, Suppl. No. 1: 96-107. DOI: http://dx.doi.org/10.1080/02827581.2013.851277

Ray, D., Bathgate, S., Moseley, D., Taylor, P., Nicoll, B., Pizzirani, S. and Gardiner, B., (2015). Comparing the provision of ecosystem services in plantation forests under alternative climate change adaptation management options in Wales. Regional Environmental Change, 15(8), pp.1501-1513.

Reyer CPO, P Lasch-Born, F Suckow, M Gutsch, A Murawski, T Pilz (2014). Projections of regional changes in forest net primary productivity for different tree species in Europe driven by climate change and carbon dioxide. Annals of Forest Science 71:211-225 DOI 10.1007/s13595-013-0306-8

Richards, F.J. 1959. A flexible growth function for empirical use. J. Exp. Bot: 290-300.

Seidl, R., Lexer, M.J., Jäger, D. and Hönninger, K. 2005. Evaluating the accuracy and generality of a hybrid forest patch model. Tree Physiology, 25, 939-951.

Seidl, R., P. Baier, W. Rammer, A. Schopf, and M. J. Lexer. 2007. Modelling tree mortality by bark beetle infestation in Norway spruce forests. Ecological Modelling 206:383–399. doi: 16/j.ecolmodel.2007.04.002.

Schelhaas, M.J., Kramer, K., Peltola, H., van der Werf, D.C., Wijdeven, S.M.J., 2007. Introducing tree interactions in wind damage simulation. Ecological Modelling 207, 197-209.

Schelhaas, M.J., 2008. The wind stability of different silvicultural systems for Douglas-fir in The Netherlands: a model-based approach. Forestry 81(3), 399-414.

Schelhaas, M.J., Clerkx, A.P.P.M., Daamen, W.P., Oldenburger, J.F., Velema, G., Schnitger, P., Schoonderwoerd, H., Kramer, H., 2014. Zesde Nederlandse Bosinventarisatie; Methoden en basisresultaten. Alterra rapport 2545, Wageningen.

Schumacher, S, H Bugmann, und D Mladenoff. „Improving the formulation of tree growth and succession in a spatially explicit landscape model“. Ecological Modelling 180, Nr. 1 (Dezember 2004): 175–94. doi:10.1016/j.ecolmodel.2003.12.055.

Schumacher, S., B. Reineking, J. Sibold, and H. Bugmann. 2006. Modeling the Impact of Climate and Vegetation on Fire Regimes in Mountain Landscapes. Landscape Ecology 21:539–554. doi: 10.1007/s10980-005-2165-7.

Schumacher, S., and H. Bugmann. 2006. The relative importance of climatic effects, wildfires and management for future forest landscape dynamics in the Swiss Alps. Global Change Biology 12:1435–1450. doi: 10.1111/j.1365-2486.2006.01188.x.

Soares P.M.M., Cardoso R.M., Miranda P.M.A., Medeiros J., Belo-Pereira M., Espírito-Santo F. 2012. WRF high resolution dynamical downscaling of ERA-Interim for Portugal. Climate Dynamics 39 (9-10): 2497-2522.

Spiecker, H., J. Hansen, and E. Klimo. 2004. Norway Spruce Conversion: Options and Consequences. EFI, Brill, Leiden, Boston, Köln.

Temperli, Christian, Harald Bugmann, und Ché Elkin. „Cross-scale interactions among bark beetles, climate change, and wind disturbances: a landscape modeling approach“. Ecological Monographs 83, Nr. 3 (2. April 2013): 383–402. doi:10.1890/12-1503.1.

Temperli, C., H. Bugmann, and C. Elkin. 2012. Adaptive management for competing forest goods and services under climate change. Ecological Applications 22:2065–2077. doi: 10.1890/12-0210.1.

Tomé, M., Faias, S. P., Tomé, J., Cortiçada, A., Soares, P. & Araújo, C., 2004. Hybridizing a stand level process-based model with growth and yield models for Eucalyptus globulus plantations in Portugal. In: Borralho, N. M. G., Pereira, J. S., Marques, C., Coutinho, J., Madeira, M. & Tomé, M. (ed.), Eucalyptus in a changing world. Proc. Iufro Conf., Aveiro, 11-15 Oct. (RAIZ, Instituto deInvestigação da Floresta e do Papel, Portugal), pp.290-297.

Wermelinger, B., and M. Seifert. 1999. Temperature-dependent reproduction of the spruce bark beetle Ips typographus, and analysis of the potential population growth. Ecological Entomology 24:103–110. doi: 10.1046/j.1365-2311.1999.00175.x.

Yousefpour R. & Hanewinkel M., Balancing Decisions for the Conversion of Norway Spruce (Picea abies L. Karst) Monocultures in the Black Forest Area of Germany, Forest Science 60: 73-84
Zimmermann, N.E. 2010. D2.2 Climate and Land Use Data. Deliverable MOTIVE project, Models for Adaptive Forest Management, FP 7 Project no. 22564.

Zubizarreta-Gerendiain A., Pukkala T., Kellomäki S., Garcia-Gonzalo J., Ikonen V-P., Peltola H. 2015. Effects of climate change on optimised stand management in the boreal forests of central Finland. European Journal of Forest Research 134(2): 273-280. DOI: 10.1007/s10342-014-0849-8.

Zubizarreta-Gerendiain, A., Pukkala, T., and Peltola, H. 2016. Effects of wood harvesting and utilisation policies on the carbon balance of forestry under changing climate: A Finnish case study. For. Pol. Econ. 62: 168–176. doi:10.1016/j.forpol.2015.08.007.
